# Supplementary material for: Research on coupling coordination and influencing factors between Urban low-carbon economy efficiency and digital finance—Evidence from 100 cities in China’s Yangtze River economic belt
Source: PLoS One. 2022 Jul 29;17(7):e0271455. doi: 10.1371/journal.pone.0271455 (PMC9337701; doi:10.1371/journal.pone.0271455)
Supplement: S1 Table — (DOCX) [file pone.0271455.s001.docx]

**S1 Table. Urban low-carbon economy efficiency and decomposition index from 2012 to 2019.**

| **Period** | **City** | **ULEE(t-1, t)** | **PEC(t-1, t)** | **SEC(t-1, t)** | **TC(t-1, t)** |
| --- | --- | --- | --- | --- | --- |
| 2012 | Anqing | 1.023182027 | 1.013525445 | 0.98964754 | 1.020088137 |
| 2012 | Anshun | 1.089622426 | 1 | 1.018939688 | 1.069368913 |
| 2012 | Bazhong | 1.065391484 | 1 | 0.950508026 | 1.120865321 |
| 2012 | Bengbu | 0.996354847 | 0.983396105 | 1.007115917 | 1.006018794 |
| 2012 | Baoshan | 1.124735494 | 1 | 1.073128259 | 1.048090464 |
| 2012 | Bozhou | 1.018466683 | 0.976837762 | 1.011497758 | 1.030764523 |
| 2012 | Changde | 1.005659671 | 1 | 1 | 1.005659671 |
| 2012 | Changzhou | 1.029189678 | 1.061634785 | 0.997393597 | 0.971971892 |
| 2012 | Chenzhou | 1.003314058 | 0.974557054 | 0.98549025 | 1.044665606 |
| 2012 | Chengdu | 1.028422039 | 1.158863007 | 0.895203614 | 0.991328172 |
| 2012 | Chizhou | 1.036162525 | 1 | 0.984944198 | 1.052001248 |
| 2012 | Chuzhou | 1.010855099 | 0.919037261 | 1.008609256 | 1.090517997 |
| 2012 | Dazhou | 0.997491924 | 1.055395149 | 0.919982033 | 1.027341776 |
| 2012 | Deyang | 1.031490455 | 1.030114224 | 0.995999472 | 1.005357962 |
| 2012 | Ezhou | 1.033261373 | 1 | 1.03253154 | 1.000706839 |
| 2012 | Fuzhou | 0.987035921 | 0.967414445 | 1.011700703 | 1.008482435 |
| 2012 | Fuyang | 1.017588451 | 1.000957248 | 0.998178795 | 1.018470141 |
| 2012 | Ganzhou | 0.959273785 | 0.992920324 | 1.000260727 | 0.96586173 |
| 2012 | Guangyuan | 1.085816088 | 1.012775789 | 1.083771008 | 0.989248573 |
| 2012 | Guiyang | 1.037646397 | 1.049309852 | 0.995740254 | 0.99311506 |
| 2012 | Hangzhou | 1.026794911 | 1.101378893 | 0.928476847 | 1.004097486 |
| 2012 | Hefei | 0.992396306 | 1.004919772 | 0.988969111 | 0.998552769 |
| 2012 | Hengyang | 1.004766481 | 1.020046456 | 0.995692685 | 0.989281462 |
| 2012 | Huzhou | 0.996649201 | 1.000161133 | 0.998673949 | 0.997811783 |
| 2012 | Huaihua | 1.063590598 | 1.236966271 | 1.003993419 | 0.856417959 |
| 2012 | Huai'an | 1.01129749 | 1.011387989 | 0.989433108 | 1.010589308 |
| 2012 | Huaibei | 1.014405019 | 1.006169675 | 1.017562218 | 0.990784473 |
| 2012 | Huainan | 0.974770741 | 0.968987143 | 1.019471675 | 0.986754933 |
| 2012 | Huanggang | 0.994409554 | 1.019142923 | 1.012225691 | 0.963946297 |
| 2012 | huangshan | 1.021319355 | 0.918734778 | 1.08926756 | 1.020555989 |
| 2012 | Huangshi | 1.035982241 | 1.041729371 | 1.006709954 | 0.987854628 |
| 2012 | Ji'an | 1.023731567 | 1 | 1 | 1.023731567 |
| 2012 | Jiaxing | 1.001788228 | 1.253263497 | 0.803281811 | 0.99509742 |
| 2012 | Jinhua | 1 | 1 | 1 | 1 |
| 2012 | Jingmen | 1.031027632 | 1.036247265 | 0.996643723 | 0.998313562 |
| 2012 | Jingzhou | 0.974024969 | 0.965248656 | 1.001573273 | 1.007507198 |
| 2012 | Jingdezhen | 0.984205354 | 1.00268391 | 0.986444148 | 0.99505979 |
| 2012 | Jiujiang | 0.93792997 | 0.962297573 | 0.997423261 | 0.977195662 |
| 2012 | Kunming | 0.921388846 | 0.885032967 | 1.032075387 | 1.008723367 |
| 2012 | Leshan | 1.030336936 | 1.033760343 | 0.995963591 | 1.000727741 |
| 2012 | Lijiang | 0.972476355 | 1 | 1.064526857 | 0.913529188 |
| 2012 | Lianyungang | 1.016733467 | 1.010785653 | 0.994914816 | 1.011025599 |
| 2012 | Liuan | 0.990386857 | 1.011828344 | 1.002171407 | 0.976688377 |
| 2012 | Liupanshui | 0.962663087 | 0.955673018 | 1.009492405 | 0.997842366 |
| 2012 | Loudi | 0.997198282 | 1.098950776 | 1.009498397 | 0.898871576 |
| 2012 | Luzhou | 1.005498375 | 1.025868428 | 1.001832386 | 0.978350885 |
| 2012 | Meishan | 1.040214664 | 1.011692867 | 1.022973646 | 1.005101309 |
| 2012 | Mianyang | 0.996415396 | 1.000237084 | 1.000918604 | 0.995264964 |
| 2012 | Nanchang | 1.002525136 | 0.997462766 | 1.005979888 | 0.999100737 |
| 2012 | Nanchong | 1.008671704 | 1.010842856 | 0.979619586 | 1.018611868 |
| 2012 | Nanjing | 1.019379975 | 1.070860756 | 0.96044265 | 0.991132364 |
| 2012 | Nantong | 1 | 1 | 1 | 1 |
| 2012 | Neijiang | 1.004422603 | 1.06982037 | 0.953522267 | 0.984633879 |
| 2012 | Ningbo | 1.001045566 | 1.087543337 | 0.911681408 | 1.009634485 |
| 2012 | Panzhihua | 1.034590666 | 1.037862901 | 1.013387725 | 0.983677932 |
| 2012 | Pingxiang | 1.014820948 | 0.997380196 | 1.046550095 | 0.972229202 |
| 2012 | Qujing | 1.484247922 | 1 | 1 | 1.484247922 |
| 2012 | Quzhou | 0.985199323 | 0.990490959 | 0.990509922 | 1.004187379 |
| 2012 | Shanghai | 1.018667506 | 1 | 1 | 1.018667506 |
| 2012 | Shangrao | 1.044978838 | 1 | 1 | 1.044978838 |
| 2012 | Shaoyang | 0.92833913 | 0.899615223 | 0.954922201 | 1.08064207 |
| 2012 | Shaoxing | 1 | 1 | 1 | 1 |
| 2012 | Shiyan | 1.023046226 | 1.025371303 | 0.997794719 | 0.999937597 |
| 2012 | Suzhou | 1 | 1 | 1 | 1 |
| 2012 | Suqian | 1.018133096 | 0.974781155 | 1.008592901 | 1.035574919 |
| 2012 | Suzhou | 0.96528176 | 0.97882487 | 1.017874038 | 0.968846707 |
| 2012 | Suizhou | 1.042539945 | 1 | 1.121395008 | 0.929681279 |
| 2012 | Taizhou | 0.999657136 | 0.975820364 | 1.016062744 | 1.008232438 |
| 2012 | Taizhou | 1 | 1 | 1 | 1 |
| 2012 | Tongling | 0.940325653 | 0.711872181 | 1.349879926 | 0.978545724 |
| 2012 | Wenzhou | 1.02036292 | 1.001014259 | 1.030457549 | 0.989200436 |
| 2012 | wuxi | 1.059222729 | 1 | 1 | 1.059222729 |
| 2012 | Wuhu | 0.976990061 | 0.979885327 | 0.992208515 | 1.004874768 |
| 2012 | Wuhan | 1.032617197 | 1.117097719 | 0.930328399 | 0.993600749 |
| 2012 | Xianning | 0.940075087 | 0.906591094 | 1.027741501 | 1.008944324 |
| 2012 | Xiangtan | 1.026926494 | 1.033428379 | 1.00293219 | 0.990803208 |
| 2012 | Xiaogan | 0.807250874 | 1 | 0.891801979 | 0.905190718 |
| 2012 | Xinyu | 1.018384287 | 1 | 1.05138379 | 0.968613267 |
| 2012 | Xuzhou | 0.998516433 | 0.992946477 | 1.014778468 | 0.990964585 |
| 2012 | Xuancheng | 0.943491189 | 0.968751933 | 0.995782653 | 0.978049218 |
| 2012 | Yancheng | 1.025424401 | 1 | 1 | 1.025424401 |
| 2012 | Yangzhou | 0.975225144 | 1.056930485 | 0.94392184 | 0.977512751 |
| 2012 | Yibin | 1.008162764 | 1.030938268 | 1.003576394 | 0.974423063 |
| 2012 | Yichang | 1.01482654 | 1.033752489 | 0.996646218 | 0.984995452 |
| 2012 | Yichun | 0.987717945 | 1.014292442 | 1.008012882 | 0.966059048 |
| 2012 | Yiyang | 0.993096454 | 1.000949805 | 1.006345962 | 0.985897632 |
| 2012 | Yingtan | 0.976109154 | 1 | 1.06097721 | 0.92000954 |
| 2012 | Yongzhou | 0.992814517 | 1.016348496 | 0.998513212 | 0.9782991 |
| 2012 | Yuxi | 1.012447186 | 1 | 1 | 1.012447186 |
| 2012 | yueyang | 1.037107877 | 1.085619139 | 0.965907248 | 0.989033534 |
| 2012 | Zhangjiajie | 0.998450368 | 1 | 0.995016631 | 1.003450935 |
| 2012 | Changsha | 0.9929957 | 1 | 1.037776721 | 0.95684908 |
| 2012 | Zhaotong | 0.854778507 | 1 | 0.797095801 | 1.072366089 |
| 2012 | Zhenjiang | 1.02215668 | 1 | 1.019242885 | 1.002858783 |
| 2012 | Chongqing | 0.947953497 | 1.168663996 | 0.79849049 | 1.015845405 |
| 2012 | Zhoushan | 1.011507445 | 0.970582717 | 1.016525285 | 1.025223009 |
| 2012 | Zhuzhou | 0.995829116 | 0.999101922 | 1.00165651 | 0.995075899 |
| 2012 | Ziyang | 1.093575586 | 1 | 1.017063188 | 1.075228755 |
| 2012 | Zigong | 1.023516445 | 0.900401684 | 1.157724857 | 0.98186814 |
| 2012 | Zunyi | 0.99506771 | 1.033533331 | 1.004119966 | 0.958832054 |
| 2013 | Anqing | 0.942410144 | 0.946456001 | 1.008975339 | 0.986867783 |
| 2013 | Anshun | 0.948966809 | 1 | 1.031974333 | 0.919564352 |
| 2013 | bazhong | 0.841572678 | 0.765021026 | 1.204471751 | 0.913317202 |
| 2013 | Bengbu | 0.990325414 | 1.013711201 | 0.992514025 | 0.98429896 |
| 2013 | Baoshan | 1.027505979 | 1 | 1.08435721 | 0.947571491 |
| 2013 | Bozhou | 0.948344881 | 0.964143072 | 1.026031012 | 0.958659393 |
| 2013 | Changde | 0.898931432 | 1 | 1 | 0.898931432 |
| 2013 | Changzhou | 0.979270752 | 0.997453802 | 0.99755348 | 0.984178347 |
| 2013 | chenzhou | 0.977028817 | 1.13503126 | 1.016845431 | 0.846534396 |
| 2013 | Chengdu | 1.365591658 | 1.080622955 | 1.251202664 | 1.009994494 |
| 2013 | Chizhou | 0.930250888 | 0.754566792 | 1.302279446 | 0.946669138 |
| 2013 | Chuzhou | 0.99278915 | 1.061390972 | 0.987798336 | 0.946920116 |
| 2013 | Dazhou | 1.018862011 | 1 | 1.037363555 | 0.982164841 |
| 2013 | Deyang | 0.871530592 | 0.972351466 | 1.003011861 | 0.893620852 |
| 2013 | Ezhou | 1.01377487 | 1 | 1.051679244 | 0.963958237 |
| 2013 | Fuzhou | 1.005917463 | 1.010067029 | 1.010577812 | 0.985467699 |
| 2013 | Fuyang | 1.017083501 | 0.998778827 | 1.000862596 | 1.017449407 |
| 2013 | Ganzhou | 1.010718496 | 1.03453025 | 0.996922767 | 0.979998712 |
| 2013 | Guangyuan | 0.97839394 | 1.056828603 | 0.961615348 | 0.962737323 |
| 2013 | Guiyang | 1.215188819 | 1.258444809 | 1.002717848 | 0.963010108 |
| 2013 | Hangzhou | 1.000208788 | 1.000284699 | 1.038536074 | 0.962820779 |
| 2013 | Hefei | 1.00714986 | 1.008011184 | 1.041212672 | 0.959597928 |
| 2013 | Hengyang | 0.99139823 | 1.018221365 | 0.995298598 | 0.978256048 |
| 2013 | Huzhou | 1.018252878 | 1.207978932 | 0.89072645 | 0.94635033 |
| 2013 | Huaihua | 1.000943357 | 1.028539301 | 1.040965215 | 0.934872519 |
| 2013 | Huai'an | 0.978772408 | 0.99547439 | 0.995028442 | 0.988134657 |
| 2013 | Huaibei | 1.007484072 | 1.06896768 | 0.992589214 | 0.949519882 |
| 2013 | Huainan | 0.982209701 | 1.00135518 | 1.014232061 | 0.967116373 |
| 2013 | Huanggang | 1.007864611 | 1 | 1 | 1.007864611 |
| 2013 | huangshan | 0.994975617 | 1.079412464 | 0.934365584 | 0.986525183 |
| 2013 | Huangshi | 1.012739387 | 1.047720571 | 0.992162407 | 0.974247863 |
| 2013 | Ji'an | 1.004369405 | 0.957837386 | 0.988208197 | 1.061092485 |
| 2013 | Jiaxing | 1.002840041 | 1 | 1.245317755 | 0.805288479 |
| 2013 | Jinhua | 0.949468046 | 1 | 1 | 0.949468046 |
| 2013 | Jingmen | 1.017877364 | 1.073182451 | 1.003982965 | 0.944703555 |
| 2013 | Jingzhou | 1.017766514 | 1.058771222 | 0.995513143 | 0.965603945 |
| 2013 | Jingdezhen | 1.013748586 | 1.054389272 | 1.019566671 | 0.943004256 |
| 2013 | Jiujiang | 0.991765314 | 1.020979637 | 0.988824341 | 0.98236456 |
| 2013 | Kunming | 1.308695175 | 1.349530603 | 1.062337065 | 0.912837409 |
| 2013 | Leshan | 0.934496159 | 0.982734496 | 1.018121215 | 0.933989152 |
| 2013 | Lijiang | 1.358754991 | 1 | 1.298078101 | 1.046743636 |
| 2013 | Lianyungang | 0.971684102 | 0.982594073 | 0.994720745 | 0.994145113 |
| 2013 | Liuan | 0.969354314 | 0.964734014 | 1.006132507 | 0.998664876 |
| 2013 | Liupanshui | 1.031885264 | 1.049837876 | 1.004314049 | 0.97867757 |
| 2013 | Loudi | 0.950774975 | 0.878149154 | 1.036345875 | 1.044731603 |
| 2013 | Luzhou | 1.037274541 | 1.036638671 | 0.995495018 | 1.00514154 |
| 2013 | Meishan | 0.964055684 | 1.012375017 | 0.996835327 | 0.955294505 |
| 2013 | Mianyang | 1.07610118 | 1.129099593 | 0.977860191 | 0.974639684 |
| 2013 | Nanchang | 0.960698369 | 1.008456948 | 1.004963558 | 0.947936786 |
| 2013 | Nanchong | 1.177676717 | 1.154113158 | 0.990070282 | 1.030651102 |
| 2013 | Nanjing | 0.975902431 | 0.992046365 | 1.009606167 | 0.974366704 |
| 2013 | Nantong | 0.768081958 | 1 | 0.832399577 | 0.922732278 |
| 2013 | Neijiang | 0.925372304 | 0.925932406 | 1.074861619 | 0.929789544 |
| 2013 | Ningbo | 1.001308816 | 1.023317078 | 0.994965783 | 0.983444085 |
| 2013 | Panzhihua | 0.897055457 | 0.905485006 | 1.067677007 | 0.927893513 |
| 2013 | Pingxiang | 0.961471029 | 0.974476362 | 0.984303869 | 1.002387637 |
| 2013 | Qujing | 1 | 1 | 1 | 1 |
| 2013 | Quzhou | 0.989949841 | 1.093271218 | 0.959310002 | 0.943900688 |
| 2013 | Shanghai | 1.023844644 | 1 | 1 | 1.023844644 |
| 2013 | Shangrao | 0.981743073 | 1 | 1 | 0.981743073 |
| 2013 | Shaoyang | 0.941635581 | 0.925412367 | 1.021621582 | 0.99599579 |
| 2013 | Shaoxing | 0.802812352 | 1 | 1 | 0.802812352 |
| 2013 | Shiyan | 1.026469993 | 1.051786353 | 1.014430246 | 0.962047549 |
| 2013 | Suzhou | 0.863493958 | 1 | 1 | 0.863493958 |
| 2013 | Suqian | 0.894676474 | 0.895888706 | 1.004355458 | 0.994316192 |
| 2013 | Suzhou | 0.985197799 | 0.998083231 | 1.010007824 | 0.977309085 |
| 2013 | Suizhou | 0.974323105 | 1 | 1.000777912 | 0.973565757 |
| 2013 | Taizhou | 0.991813959 | 1.23701285 | 0.865014133 | 0.926899829 |
| 2013 | Taizhou | 0.772552533 | 0.843100621 | 0.993463123 | 0.922352355 |
| 2013 | Tongling | 0.999986611 | 1.004164076 | 1.011044128 | 0.984961814 |
| 2013 | Wenzhou | 1.016026001 | 1.115265499 | 0.955741892 | 0.953204168 |
| 2013 | wuxi | 0.906192872 | 1 | 1 | 0.906192872 |
| 2013 | Wuhu | 1.004942328 | 0.996424531 | 1.009541026 | 0.999016717 |
| 2013 | Wuhan | 0.988839804 | 1.015466065 | 1.003202577 | 0.970670622 |
| 2013 | Xianning | 1.017102117 | 1.01044628 | 1.010678735 | 0.995951525 |
| 2013 | Xiangtan | 0.979614085 | 1.003013739 | 1.001421074 | 0.975284703 |
| 2013 | Xiaogan | 0.994190866 | 0.91945727 | 1.113139562 | 0.971378752 |
| 2013 | Xinyu | 0.973029178 | 1 | 1.31959682 | 0.737368538 |
| 2013 | Xuzhou | 0.974231462 | 1.321622291 | 0.751340236 | 0.981111009 |
| 2013 | Xuancheng | 0.984153693 | 0.977657971 | 1.013948433 | 0.992796214 |
| 2013 | Yancheng | 0.9297438 | 1 | 1 | 0.9297438 |
| 2013 | Yangzhou | 0.876869701 | 0.825817122 | 1.106012099 | 0.960044365 |
| 2013 | Yibin | 1.127252636 | 1.192235093 | 0.972373167 | 0.97235845 |
| 2013 | Yichang | 0.994696267 | 1.051958934 | 0.991515857 | 0.953656642 |
| 2013 | Yichun | 0.98221435 | 0.977609788 | 0.998062812 | 1.00666011 |
| 2013 | Yiyang | 0.985794304 | 1.000522624 | 1.008012505 | 0.977447571 |
| 2013 | Yingtan | 1.015015431 | 1 | 1.033260511 | 0.982342227 |
| 2013 | yongzhou | 0.998644674 | 1.032574794 | 0.999331873 | 0.967786883 |
| 2013 | Yuxi | 1.04751573 | 1 | 1 | 1.04751573 |
| 2013 | yueyang | 0.977453151 | 1.08916222 | 1.037446678 | 0.865042805 |
| 2013 | Zhangjiajie | 1.030710978 | 1 | 0.994861 | 1.036035162 |
| 2013 | Changsha | 1.029111725 | 1 | 1.015167426 | 1.01373596 |
| 2013 | Zhaotong | 1.155703223 | 1 | 1.243516792 | 0.929382885 |
| 2013 | Zhenjiang | 0.95185423 | 1 | 1.077776061 | 0.883165125 |
| 2013 | Chongqing | 0.919000143 | 0.991167924 | 1.018827413 | 0.910055164 |
| 2013 | Zhoushan | 1.012552046 | 1.134944443 | 0.908334079 | 0.982193701 |
| 2013 | Zhuzhou | 0.996000237 | 1.029623468 | 0.998099292 | 0.969186289 |
| 2013 | Ziyang | 0.881561977 | 1 | 0.961857905 | 0.916519969 |
| 2013 | Zigong | 1.069946977 | 1.110615426 | 1.018352512 | 0.946020213 |
| 2013 | Zunyi | 1.361866577 | 1.324985397 | 1.001687568 | 1.02610354 |
| 2014 | Anqing | 1.010486507 | 1.001388233 | 1.003308083 | 1.005758529 |
| 2014 | Anshun | 1.041895244 | 0.725837598 | 1.391542189 | 1.031545092 |
| 2014 | bazhong | 0.93521499 | 0.983116907 | 0.957477981 | 0.993522025 |
| 2014 | Bengbu | 1.024469162 | 1.016508058 | 1.018570934 | 0.989456681 |
| 2014 | Baoshan | 1.008009841 | 1 | 0.992699619 | 1.015422814 |
| 2014 | Bozhou | 1.034180403 | 0.995266694 | 1.008998516 | 1.029831818 |
| 2014 | Changde | 0.986779113 | 1 | 1 | 0.986779113 |
| 2014 | Changzhou | 1.063281689 | 1.248191904 | 1.010321735 | 0.843154723 |
| 2014 | chenzhou | 1.040394143 | 1 | 1 | 1.040394143 |
| 2014 | Chengdu | 0.741137502 | 1 | 0.784902633 | 0.944241326 |
| 2014 | Chizhou | 1.033308821 | 0.99502586 | 1.004920844 | 1.033389192 |
| 2014 | Chuzhou | 1.035407961 | 1.024104737 | 0.999887802 | 1.011150624 |
| 2014 | Dazhou | 0.95967658 | 0.716765343 | 1.223181769 | 1.094603668 |
| 2014 | Deyang | 1.1697124 | 1.110305213 | 0.992548627 | 1.06141427 |
| 2014 | Ezhou | 1.012756112 | 1 | 1.021931062 | 0.991021948 |
| 2014 | Fuzhou | 1.025481767 | 1.009214929 | 0.992374471 | 1.023926289 |
| 2014 | Fuyang | 1.04311874 | 1.067666693 | 1.015245968 | 0.962336104 |
| 2014 | Ganzhou | 0.935189072 | 0.863302182 | 0.98841941 | 1.095961555 |
| 2014 | Guangyuan | 1.035031876 | 1.004855631 | 0.99999011 | 1.030040616 |
| 2014 | Guiyang | 0.918033933 | 0.914431847 | 1.001746813 | 1.002188517 |
| 2014 | Hangzhou | 1.001565371 | 0.971672968 | 1.044423564 | 0.986921291 |
| 2014 | Hefei | 1.004790757 | 0.987260391 | 1.003554176 | 1.014152103 |
| 2014 | Hengyang | 1.007819721 | 1.029490387 | 1.00465554 | 0.974413682 |
| 2014 | Huzhou | 0.974180422 | 0.852269679 | 1.120686567 | 1.019948381 |
| 2014 | Huaihua | 1.04382209 | 0.974566554 | 0.990407535 | 1.081436554 |
| 2014 | Huai'an | 1.012605126 | 1.020261884 | 1.001918444 | 0.9905949 |
| 2014 | Huaibei | 1.028257301 | 1.007677878 | 1.004488157 | 1.015863268 |
| 2014 | Huainan | 1.024480256 | 1.034979374 | 0.982216542 | 1.007777491 |
| 2014 | Huanggang | 1.051034516 | 1 | 1 | 1.051034516 |
| 2014 | huangshan | 1.042135496 | 1.030166066 | 0.984464441 | 1.027583009 |
| 2014 | Huangshi | 1.009050799 | 0.992777213 | 1.008183605 | 1.008141747 |
| 2014 | Ji'an | 0.96587511 | 0.845827618 | 0.990353559 | 1.153051875 |
| 2014 | Jiaxing | 1.013964053 | 1 | 1 | 1.013964053 |
| 2014 | Jinhua | 1.053221332 | 1 | 1 | 1.053221332 |
| 2014 | Jingmen | 0.988228264 | 0.959496817 | 1.013141297 | 1.016585039 |
| 2014 | Jingzhou | 1.01721787 | 1.014782327 | 0.987917245 | 1.014659952 |
| 2014 | Jingdezhen | 0.993023728 | 0.960855822 | 1.019995027 | 1.013219048 |
| 2014 | Jiujiang | 1.007017704 | 0.987714349 | 1.003031841 | 1.016461709 |
| 2014 | Kunming | 0.890255484 | 0.890214533 | 0.95476888 | 1.047422073 |
| 2014 | Leshan | 1.086366089 | 1.043579335 | 0.993058149 | 1.048276983 |
| 2014 | Lijiang | 0.942980262 | 1 | 0.883107054 | 1.067798358 |
| 2014 | Lianyungang | 0.979898276 | 0.927417035 | 1.000039443 | 1.056546937 |
| 2014 | Liuan | 1.266085366 | 1.226803571 | 0.994586242 | 1.037637145 |
| 2014 | Liupanshui | 1.110143725 | 1.062934416 | 1.005428344 | 1.038775302 |
| 2014 | Loudi | 1.015718992 | 1.003227575 | 1.004201599 | 1.008215114 |
| 2014 | Luzhou | 0.934062648 | 0.956359809 | 1.009969656 | 0.967044285 |
| 2014 | Meishan | 1.118388457 | 1.163651078 | 0.99625196 | 0.964718731 |
| 2014 | Mianyang | 0.91070138 | 0.892668361 | 1.022415153 | 0.997834637 |
| 2014 | Nanchang | 1.051067347 | 1.0666201 | 0.985973164 | 0.999437606 |
| 2014 | Nanchong | 0.829631564 | 0.84451589 | 1.034007048 | 0.950066362 |
| 2014 | Nanjing | 1.016712076 | 1.005835317 | 1.017353108 | 0.993572094 |
| 2014 | Nantong | 0.985296064 | 0.9052329 | 1.069915224 | 1.017318754 |
| 2014 | Neijiang | 1.080370004 | 1.076393493 | 0.95292492 | 1.05327741 |
| 2014 | Ningbo | 1.010018832 | 0.991757161 | 1.019032579 | 0.999392435 |
| 2014 | Panzhihua | 1.136770705 | 1.141081612 | 0.93684345 | 1.0633816 |
| 2014 | Pingxiang | 1.046466542 | 1.012462456 | 1.016209038 | 1.017099326 |
| 2014 | Qujing | 1 | 1 | 1 | 1 |
| 2014 | Quzhou | 1.000258767 | 0.925157515 | 1.042154283 | 1.037444011 |
| 2014 | Shanghai | 1.04766908 | 1 | 1 | 1.04766908 |
| 2014 | Shangrao | 1.059554313 | 1 | 1 | 1.059554313 |
| 2014 | Shaoyang | 1.026345323 | 0.97171722 | 1.015181252 | 1.04042318 |
| 2014 | Shaoxing | 1.002718068 | 1 | 1 | 1.002718068 |
| 2014 | Shiyan | 0.999859812 | 0.981217036 | 0.999695897 | 1.01930962 |
| 2014 | Suzhou | 1.026851213 | 1 | 1 | 1.026851213 |
| 2014 | Suqian | 1.021724495 | 1.01565739 | 0.997801684 | 1.008189893 |
| 2014 | Suzhou | 1.013782287 | 1.005567484 | 1.000255814 | 1.007911483 |
| 2014 | Suizhou | 1.043370278 | 1 | 1.080144193 | 0.965954624 |
| 2014 | Taizhou | 0.996182068 | 0.823400606 | 1.159703363 | 1.043231329 |
| 2014 | Taizhou | 1.026397073 | 1.016078241 | 1.001226584 | 1.008918026 |
| 2014 | Tongling | 1.00982285 | 0.986968365 | 1.008157545 | 1.014877341 |
| 2014 | Wenzhou | 1.005845873 | 1.129682005 | 1.069369592 | 0.83262107 |
| 2014 | wuxi | 0.997518694 | 1 | 1 | 0.997518694 |
| 2014 | Wuhu | 1.008572699 | 1.01463161 | 1.000064734 | 0.993964119 |
| 2014 | Wuhan | 1.033548036 | 1.026900138 | 1.004372242 | 1.002092363 |
| 2014 | Xianning | 0.999459779 | 0.95695317 | 1.015584634 | 1.028391586 |
| 2014 | Xiangtan | 0.98088661 | 0.987697482 | 1.003216804 | 0.989919915 |
| 2014 | Xiaogan | 0.991767968 | 0.905231849 | 1.00055846 | 1.094984025 |
| 2014 | Xinyu | 1.008516486 | 1 | 1 | 1.008516486 |
| 2014 | Xuzhou | 1.024319966 | 0.859653082 | 1.180117347 | 1.009688057 |
| 2014 | Xuancheng | 1.184968613 | 1.370471269 | 0.942650375 | 0.917246931 |
| 2014 | Yancheng | 1.018816223 | 1 | 1 | 1.018816223 |
| 2014 | Yangzhou | 1.015419058 | 1.210921853 | 1.002344872 | 0.836588751 |
| 2014 | Yibin | 0.896370375 | 0.895303392 | 1.034483727 | 0.967817791 |
| 2014 | Yichang | 1.004250807 | 1.022804609 | 0.975985859 | 1.006018548 |
| 2014 | Yichun | 0.999490665 | 0.97253874 | 1.005238911 | 1.02235692 |
| 2014 | Yiyang | 0.995837964 | 0.980555737 | 1.016121483 | 0.999472296 |
| 2014 | Yingtan | 1.012220879 | 1 | 1 | 1.012220879 |
| 2014 | yongzhou | 1.021011821 | 0.974217945 | 1.018998797 | 1.028492133 |
| 2014 | Yuxi | 0.96854062 | 1 | 1 | 0.96854062 |
| 2014 | yueyang | 1.026295304 | 1.074172807 | 0.932058792 | 1.025073202 |
| 2014 | Zhangjiajie | 1.040478955 | 1 | 1.03869031 | 1.00172202 |
| 2014 | Changsha | 1.003184509 | 1 | 1 | 1.003184509 |
| 2014 | Zhaotong | 0.941579349 | 1 | 0.946108174 | 0.995213206 |
| 2014 | Zhenjiang | 1.056867641 | 1 | 1.000058735 | 1.05680557 |
| 2014 | Chongqing | 1.156407929 | 1.013125714 | 1.010086628 | 1.130027731 |
| 2014 | Zhoushan | 0.939832848 | 0.854158783 | 1.086613414 | 1.012597723 |
| 2014 | Zhuzhou | 1.014031632 | 1.013910815 | 1.003030001 | 0.997097951 |
| 2014 | Ziyang | 1.110424655 | 1 | 1.039654604 | 1.068070733 |
| 2014 | Zigong | 1.005223234 | 1 | 0.994463522 | 1.010819614 |
| 2014 | Zunyi | 0.835471255 | 0.930599584 | 0.913108234 | 0.983210254 |
| 2015 | Anqing | 0.973590199 | 0.94843218 | 0.987542043 | 1.039475645 |
| 2015 | Anshun | 1.039330145 | 0.893213722 | 0.963865037 | 1.20720746 |
| 2015 | bazhong | 1.040004396 | 0.936807576 | 0.852101306 | 1.302847398 |
| 2015 | Bengbu | 1.006261277 | 0.991950631 | 0.970104446 | 1.0456882 |
| 2015 | Baoshan | 0.983261321 | 0.829092439 | 1.001238547 | 1.18448193 |
| 2015 | Bozhou | 1.020874116 | 0.886676708 | 0.956666942 | 1.203500086 |
| 2015 | Changde | 1.065275524 | 1 | 1 | 1.065275524 |
| 2015 | Changzhou | 0.981426334 | 1 | 1 | 0.981426334 |
| 2015 | chenzhou | 1.065997091 | 0.850907816 | 0.968832971 | 1.293077624 |
| 2015 | Chengdu | 0.976364212 | 1 | 0.974187019 | 1.002234882 |
| 2015 | Chizhou | 1.076574668 | 1.107910033 | 0.919002696 | 1.057359998 |
| 2015 | Chuzhou | 1.024940666 | 0.95957208 | 0.951302486 | 1.122800222 |
| 2015 | Dazhou | 0.984891733 | 0.922859787 | 0.976363237 | 1.093053328 |
| 2015 | Deyang | 1.010812189 | 1.143424272 | 0.852343256 | 1.037166627 |
| 2015 | Ezhou | 1.005368394 | 1 | 1.041355631 | 0.965441934 |
| 2015 | Fuzhou | 0.994994146 | 0.888254732 | 0.983604622 | 1.138839272 |
| 2015 | Fuyang | 0.995356098 | 0.821945331 | 0.993485542 | 1.218916617 |
| 2015 | Ganzhou | 1.024902524 | 0.872845928 | 0.973103578 | 1.206662741 |
| 2015 | Guangyuan | 1.058742775 | 0.988600605 | 1.015721204 | 1.054374924 |
| 2015 | Guiyang | 1.02651598 | 1.04847296 | 0.997089507 | 0.981915993 |
| 2015 | Hangzhou | 0.996204001 | 1.230616603 | 0.835424845 | 0.968987388 |
| 2015 | Hefei | 1.003790946 | 0.991446575 | 0.996345153 | 1.016164796 |
| 2015 | Hengyang | 1.018596491 | 0.925565809 | 0.995477496 | 1.105511909 |
| 2015 | Huzhou | 0.979241597 | 1.006750881 | 0.967108543 | 1.005755962 |
| 2015 | Huaihua | 1.051091446 | 0.824327895 | 0.93974847 | 1.356840749 |
| 2015 | Huai'an | 1.0016091 | 0.956858648 | 0.999752624 | 1.047027101 |
| 2015 | Huaibei | 0.994924295 | 1.077748194 | 0.907162165 | 1.017625084 |
| 2015 | Huainan | 1.068946154 | 1.127107137 | 0.967469848 | 0.980286881 |
| 2015 | Huanggang | 0.925474201 | 0.9752549 | 0.867355526 | 1.094079866 |
| 2015 | huangshan | 1.019447514 | 0.989979522 | 0.925903477 | 1.112174531 |
| 2015 | Huangshi | 0.949234731 | 0.951494067 | 0.984017528 | 1.013828979 |
| 2015 | Ji'an | 1.017411806 | 0.9206155 | 0.985843518 | 1.121012629 |
| 2015 | Jiaxing | 0.951974963 | 1 | 1 | 0.951974963 |
| 2015 | Jinhua | 1 | 1 | 1 | 1 |
| 2015 | Jingmen | 0.958998412 | 0.952385716 | 0.961975618 | 1.046745133 |
| 2015 | Jingzhou | 1.06079996 | 1.023259938 | 0.976187385 | 1.061975095 |
| 2015 | Jingdezhen | 0.990192092 | 0.999861998 | 0.929554955 | 1.065379464 |
| 2015 | Jiujiang | 1.014596608 | 0.887820639 | 1.004987324 | 1.13712337 |
| 2015 | Kunming | 0.967275464 | 0.916069032 | 1.030822692 | 1.024325535 |
| 2015 | Leshan | 1.008999148 | 1.004252208 | 0.980752902 | 1.024444422 |
| 2015 | Lijiang | 1.0718911 | 1 | 0.937021223 | 1.143934708 |
| 2015 | Lianyungang | 1.047302183 | 0.988015128 | 1.007469512 | 1.052147197 |
| 2015 | Liuan | 0.70957523 | 0.601019669 | 1.002216962 | 1.178007386 |
| 2015 | Liupanshui | 1.084569072 | 0.987202691 | 0.991335284 | 1.108231069 |
| 2015 | Loudi | 1.005967539 | 0.957317621 | 0.963771461 | 1.090319682 |
| 2015 | Luzhou | 1.013016372 | 0.948113958 | 0.99072384 | 1.078458184 |
| 2015 | Meishan | 1.040149172 | 1.00896678 | 0.913487992 | 1.1285373 |
| 2015 | Mianyang | 1.036941678 | 1.005696161 | 0.980802363 | 1.051250063 |
| 2015 | Nanchang | 0.998674208 | 0.965011877 | 1.020681189 | 1.013913871 |
| 2015 | Nanchong | 1.018410925 | 0.891147699 | 0.986893971 | 1.157984812 |
| 2015 | Nanjing | 1.0218411 | 1.055423086 | 0.989252232 | 0.978700338 |
| 2015 | Nantong | 1.03701125 | 1.104688086 | 1.101697236 | 0.852082288 |
| 2015 | Neijiang | 0.98325415 | 1.003343525 | 0.857179883 | 1.14325778 |
| 2015 | Ningbo | 0.966721169 | 1.015515396 | 0.921319332 | 1.033247904 |
| 2015 | Panzhihua | 1.020154049 | 1.424211587 | 0.749317028 | 0.955929009 |
| 2015 | Pingxiang | 0.998469887 | 0.997944731 | 0.967091075 | 1.03457292 |
| 2015 | Qujing | 0.79280414 | 0.812377267 | 0.97740128 | 0.998470514 |
| 2015 | Quzhou | 1.001837632 | 0.9729708 | 0.997350608 | 1.032403998 |
| 2015 | Shanghai | 1.030690808 | 1 | 1 | 1.030690808 |
| 2015 | Shangrao | 0.727399199 | 0.730219577 | 0.9893046 | 1.006906902 |
| 2015 | Shaoyang | 1.500574631 | 1.236140985 | 1.009713962 | 1.202240163 |
| 2015 | Shaoxing | 0.90100197 | 1 | 1 | 0.90100197 |
| 2015 | Shiyan | 0.99224926 | 0.888932165 | 0.988819007 | 1.128847711 |
| 2015 | Suzhou | 0.915044161 | 1 | 1 | 0.915044161 |
| 2015 | Suqian | 0.995806864 | 0.940550841 | 1.001838873 | 1.056805246 |
| 2015 | Suzhou | 0.977658038 | 0.85140872 | 0.957629649 | 1.199088664 |
| 2015 | Suizhou | 0.986154683 | 1 | 0.838375676 | 1.176268243 |
| 2015 | Taizhou | 0.970880269 | 1.054698449 | 0.997816087 | 0.922543528 |
| 2015 | Taizhou | 1.009127378 | 1.167329443 | 1.005346747 | 0.859877673 |
| 2015 | Tongling | 1.077173025 | 1.417392288 | 0.751688463 | 1.011014814 |
| 2015 | Wenzhou | 1.005412649 | 1 | 1 | 1.005412649 |
| 2015 | wuxi | 0.995673785 | 1 | 1 | 0.995673785 |
| 2015 | Wuhu | 0.99842252 | 0.965949879 | 0.996873801 | 1.036858741 |
| 2015 | Wuhan | 1.009254454 | 1.035607251 | 0.964388786 | 1.010539842 |
| 2015 | Xianning | 1.023221404 | 1.002693072 | 0.924640175 | 1.103643584 |
| 2015 | Xiangtan | 1.024787806 | 1.050397443 | 0.970405807 | 1.00537228 |
| 2015 | Xiaogan | 1.009630191 | 0.929063745 | 0.965284528 | 1.125800587 |
| 2015 | Xinyu | 1.022774267 | 1 | 1 | 1.022774267 |
| 2015 | Xuzhou | 0.999424311 | 0.983293678 | 0.987197769 | 1.029585688 |
| 2015 | Xuancheng | 1.0386433 | 1 | 0.874094726 | 1.188250276 |
| 2015 | Yancheng | 0.902383034 | 0.902819692 | 0.776636699 | 1.286980567 |
| 2015 | Yangzhou | 1.010085091 | 1 | 1 | 1.010085091 |
| 2015 | Yibin | 1.002956919 | 0.924856062 | 0.977820163 | 1.109044935 |
| 2015 | Yichang | 0.976823832 | 0.943158001 | 0.968780228 | 1.069070945 |
| 2015 | Yichun | 0.993754398 | 0.862056529 | 0.994161898 | 1.159541253 |
| 2015 | Yiyang | 1.06359491 | 1.00736979 | 0.9297897 | 1.135540417 |
| 2015 | Yingtan | 1.013669557 | 1 | 0.77126525 | 1.314294344 |
| 2015 | Yongzhou | 1.074167668 | 0.982583284 | 0.953584473 | 1.146419419 |
| 2015 | Yuxi | 1.378282103 | 1 | 1 | 1.378282103 |
| 2015 | Yueyang | 1.006392535 | 0.915043587 | 1.023195738 | 1.074897128 |
| 2015 | Zhangjiajie | 1.053774378 | 1 | 0.896852107 | 1.174970065 |
| 2015 | Changsha | 0.995781598 | 1 | 1 | 0.995781598 |
| 2015 | Zhaotong | 1.153627 | 1 | 1.066343264 | 1.08185332 |
| 2015 | Zhenjiang | 1.012914303 | 1 | 1.013421643 | 0.999499379 |
| 2015 | Chongqing | 1.004148833 | 1.05520971 | 0.94406082 | 1.007997221 |
| 2015 | Zhoushan | 0.974417394 | 0.953468716 | 0.973181265 | 1.050134291 |
| 2015 | Zhuzhou | 0.987180433 | 0.97174124 | 0.984362919 | 1.032026048 |
| 2015 | Ziyang | 1.038525516 | 1 | 0.859386648 | 1.208449675 |
| 2015 | Zigong | 0.975202337 | 1 | 0.903220569 | 1.079694563 |
| 2015 | Zunyi | 1.424631187 | 1.074576023 | 1.095160423 | 1.210563459 |
| 2016 | Anqing | 1.029979719 | 1.024962792 | 1.003351707 | 1.00153788 |
| 2016 | Anshun | 1.036758979 | 1.104031819 | 1.072573314 | 0.875526363 |
| 2016 | Bazhong | 0.944545203 | 0.922133775 | 1.38111243 | 0.741651337 |
| 2016 | Bengbu | 1.032983531 | 1.034439188 | 1.020083939 | 0.978931995 |
| 2016 | Baoshan | 1.067298438 | 1.206138126 | 1.038239392 | 0.852297718 |
| 2016 | Bozhou | 1.045788224 | 1.158596024 | 1.081409531 | 0.834682908 |
| 2016 | Changde | 1.085575245 | 1 | 1 | 1.085575245 |
| 2016 | Changzhou | 1.05009927 | 1 | 1 | 1.05009927 |
| 2016 | Chenzhou | 1.26966103 | 1.175215436 | 1.032169662 | 1.046692742 |
| 2016 | Chengdu | 1.00373522 | 0.973879655 | 1.075705848 | 0.958120959 |
| 2016 | Chizhou | 1.030049883 | 1.202163255 | 0.930929569 | 0.92040291 |
| 2016 | Chuzhou | 1.042424875 | 1.050582195 | 1.070095342 | 0.92724021 |
| 2016 | Dazhou | 1.020965795 | 1.159495669 | 1.017581116 | 0.86531259 |
| 2016 | Deyang | 1.073211762 | 1.049686275 | 1.083775845 | 0.943379507 |
| 2016 | Ezhou | 0.995739261 | 1 | 0.969564848 | 1.026996042 |
| 2016 | Fuzhou | 1.012565693 | 1.195934437 | 1.047239427 | 0.808481064 |
| 2016 | Fuyang | 1.022718462 | 1.1364482 | 1.010873919 | 0.890244828 |
| 2016 | Ganzhou | 1.061665111 | 1.287742281 | 0.999481655 | 0.824866684 |
| 2016 | Guangyuan | 1.032085747 | 1.003288647 | 1.012314774 | 1.016188575 |
| 2016 | Guiyang | 1.016141709 | 1.008759976 | 1.000431052 | 1.006883611 |
| 2016 | Hangzhou | 1.011721876 | 0.851950592 | 1.206023277 | 0.98467076 |
| 2016 | Hefei | 0.996534974 | 0.995208503 | 1.056952145 | 0.947377667 |
| 2016 | Hengyang | 1.023153847 | 1.172274762 | 0.989173989 | 0.882345832 |
| 2016 | Huzhou | 1.017240346 | 0.994373186 | 1.038630122 | 0.9849479 |
| 2016 | Huaihua | 1.048058017 | 1.244768245 | 1.074420873 | 0.783650449 |
| 2016 | Huai'an | 1.050304143 | 1.099775302 | 1.01924078 | 0.936988638 |
| 2016 | Huaibei | 1.005040224 | 0.916745969 | 1.113658798 | 0.984424202 |
| 2016 | Huainan | 0.954558717 | 0.885081273 | 1.064812183 | 1.012853154 |
| 2016 | Huanggang | 1.115149934 | 1.025372956 | 1.152929762 | 0.943297218 |
| 2016 | huangshan | 1.019241629 | 0.984050805 | 1.140134336 | 0.908455393 |
| 2016 | Huangshi | 1.035693735 | 1.0281671 | 1.015314289 | 0.992126724 |
| 2016 | Ji'an | 1.049623049 | 1.340750822 | 0.99976254 | 0.783048076 |
| 2016 | Jiaxing | 1.058256146 | 1 | 1 | 1.058256146 |
| 2016 | Jinhua | 0.790127529 | 1 | 1 | 0.790127529 |
| 2016 | Jingmen | 1.011657429 | 1.052203847 | 1.054847369 | 0.91147333 |
| 2016 | Jingzhou | 0.929705145 | 1.003420979 | 1.040968694 | 0.890070463 |
| 2016 | Jingdezhen | 1.042632069 | 1.047671304 | 1.094974124 | 0.908870848 |
| 2016 | Jiujiang | 1.059726034 | 1.23086324 | 0.9772641 | 0.880991781 |
| 2016 | Kunming | 0.979234813 | 0.974522522 | 1.0154863 | 0.989511612 |
| 2016 | Leshan | 1.034558022 | 1.020756525 | 1.026129515 | 0.987712406 |
| 2016 | Lijiang | 1.054685965 | 1 | 1.246265196 | 0.846277316 |
| 2016 | Lianyungang | 1.066583847 | 1.193305946 | 0.997965436 | 0.895628073 |
| 2016 | Liuan | 1.084777538 | 1.357731981 | 1.013801294 | 0.788086331 |
| 2016 | Liupanshui | 1.166477537 | 1.322092263 | 1.065793448 | 0.82783078 |
| 2016 | Loudi | 1.009731993 | 1.125553958 | 1.01089621 | 0.887428208 |
| 2016 | Luzhou | 1.012801233 | 1.058792966 | 1.016246457 | 0.94126981 |
| 2016 | Meishan | 0.999760919 | 0.981867779 | 1.111891032 | 0.91575842 |
| 2016 | Mianyang | 0.985708772 | 1.049669087 | 1.016131008 | 0.924158601 |
| 2016 | Nanchang | 1.008405853 | 1.055165574 | 1.000855847 | 0.954867724 |
| 2016 | Nanchong | 0.996876907 | 1.16192356 | 1.01168089 | 0.848047993 |
| 2016 | Nanjing | 1.008061427 | 0.9555271 | 1.030780463 | 1.023476346 |
| 2016 | Nantong | 1.042310396 | 1 | 1.019193212 | 1.022681846 |
| 2016 | Neijiang | 1.022116016 | 1 | 1.266065866 | 0.80731662 |
| 2016 | Ningbo | 1.019685501 | 0.919784555 | 1.152446367 | 0.961965305 |
| 2016 | Panzhihua | 1.028429744 | 1 | 1.007375444 | 1.020900152 |
| 2016 | Pingxiang | 1.03286458 | 1.018389186 | 1.03385964 | 0.980997778 |
| 2016 | Qujing | 1.101927243 | 1.230955174 | 1.02312123 | 0.874950705 |
| 2016 | Quzhou | 1.027344668 | 1.017263399 | 1.007112824 | 1.002777605 |
| 2016 | Shanghai | 1.064806226 | 1 | 1 | 1.064806226 |
| 2016 | Shangrao | 1.04055391 | 1.346607979 | 0.968278956 | 0.798036782 |
| 2016 | Shaoyang | 0.764661893 | 0.976648224 | 0.999740226 | 0.783148492 |
| 2016 | Shaoxing | 1.011971916 | 1 | 1 | 1.011971916 |
| 2016 | Shiyan | 1.01861242 | 1.166591452 | 1.014185777 | 0.860939556 |
| 2016 | Suzhou | 1.232511904 | 1 | 1 | 1.232511904 |
| 2016 | Suqian | 1.034927746 | 1.141812887 | 1.000053992 | 0.906341033 |
| 2016 | Suzhou | 1.022249978 | 1.201910509 | 1.055941184 | 0.805462357 |
| 2016 | Suizhou | 1.016845344 | 1 | 1.145334777 | 0.887814955 |
| 2016 | Taizhou | 0.996700925 | 1.151490901 | 0.922758647 | 0.938028918 |
| 2016 | Taizhou | 1.05719376 | 1 | 1 | 1.05719376 |
| 2016 | Tongling | 0.948415421 | 0.722426945 | 1.293550993 | 1.014895011 |
| 2016 | Wenzhou | 0.991700165 | 1 | 1 | 0.991700165 |
| 2016 | Wuxi | 1.0610881 | 1 | 1 | 1.0610881 |
| 2016 | Wuhu | 1.023075454 | 1.044239292 | 0.99352993 | 0.98611299 |
| 2016 | Wuhan | 1.001437696 | 0.998800654 | 1.021768364 | 0.981279363 |
| 2016 | Xianning | 1.019701674 | 1.099478756 | 1.092480118 | 0.848931694 |
| 2016 | Xiangtan | 0.999750212 | 0.966328723 | 1.034090484 | 1.000479224 |
| 2016 | Xiaogan | 0.955234733 | 1.098246851 | 1.038012975 | 0.837929261 |
| 2016 | Xinyu | 1.044871787 | 1 | 1 | 1.044871787 |
| 2016 | Xuzhou | 1.031997645 | 0.996206934 | 1.096440352 | 0.944809247 |
| 2016 | Xuancheng | 1.063919694 | 0.952307082 | 1.107300216 | 1.008942577 |
| 2016 | Yancheng | 1.034068377 | 1.107640882 | 1.110992361 | 0.840309372 |
| 2016 | Yangzhou | 1.023870924 | 1 | 1 | 1.023870924 |
| 2016 | Yibin | 1.050266543 | 1.176456288 | 1.025969499 | 0.870140305 |
| 2016 | Yichang | 1.068529947 | 1.205327514 | 1.068548572 | 0.829635562 |
| 2016 | Yichun | 1.038181615 | 1.289884762 | 1.009578433 | 0.797227658 |
| 2016 | Yiyang | 1.056176569 | 1.231511614 | 1.064150093 | 0.805925931 |
| 2016 | Yingtan | 1.018245982 | 1 | 1.296570797 | 0.785337742 |
| 2016 | Yongzhou | 1.067305341 | 1.293913123 | 1.049436322 | 0.786008919 |
| 2016 | Yuxi | 0.694801369 | 1 | 1 | 0.694801369 |
| 2016 | Yueyang | 1.052999031 | 1.092844116 | 1.049952352 | 0.917698797 |
| 2016 | Zhangjiajie | 1.042889255 | 1 | 1.387454157 | 0.75165673 |
| 2016 | Changsha | 1.037717984 | 1 | 1 | 1.037717984 |
| 2016 | Zhaotong | 0.821597026 | 1 | 1 | 0.821597026 |
| 2016 | Zhenjiang | 1.099658732 | 1 | 1.039692619 | 1.057676771 |
| 2016 | Chongqing | 1.116581488 | 1.020353812 | 1.075382425 | 1.01759906 |
| 2016 | Zhoushan | 1.051548602 | 1.127630069 | 1.042780437 | 0.894272399 |
| 2016 | Zhuzhou | 1.013323705 | 1.089981925 | 1.013556107 | 0.917236041 |
| 2016 | Ziyang | 0.926749733 | 1 | 1.163620591 | 0.796436347 |
| 2016 | Zigong | 1.0813008 | 1 | 1.338987219 | 0.807551248 |
| 2016 | Zunyi | 0.728219476 | 1 | 0.820167836 | 0.8878908 |
| 2017 | Anqing | 1.036700994 | 1.236819855 | 0.93293116 | 0.898457354 |
| 2017 | Anshun | 0.99196344 | 1.035986695 | 1.023787557 | 0.935258452 |
| 2017 | Bazhong | 0.930035891 | 1.027061701 | 1.017910187 | 0.889597827 |
| 2017 | Bengbu | 1.009960362 | 1.077631463 | 1.006974141 | 0.930712933 |
| 2017 | Baoshan | 0.850858803 | 1 | 1.117705052 | 0.761255218 |
| 2017 | Bozhou | 0.932702871 | 0.958293495 | 1.010616546 | 0.963071142 |
| 2017 | Changde | 0.884913081 | 1 | 1 | 0.884913081 |
| 2017 | Changzhou | 1.211685031 | 1 | 1 | 1.211685031 |
| 2017 | chenzhou | 0.753947678 | 1 | 1 | 0.753947678 |
| 2017 | Chengdu | 0.973408003 | 1.026820917 | 1.043207243 | 0.908719007 |
| 2017 | Chizhou | 0.950601937 | 1 | 0.923326704 | 1.029540175 |
| 2017 | Chuzhou | 0.885067399 | 0.867708958 | 0.964131748 | 1.057951794 |
| 2017 | Dazhou | 0.888231748 | 0.953016746 | 1.007916682 | 0.924700573 |
| 2017 | Deyang | 0.892040958 | 1 | 0.983806238 | 0.906724234 |
| 2017 | Ezhou | 1.032957317 | 1 | 1.148416383 | 0.899462365 |
| 2017 | Fuzhou | 0.896316077 | 0.988353511 | 0.956150902 | 0.948467464 |
| 2017 | Fuyang | 0.972540572 | 1.009701397 | 1.015087329 | 0.948880155 |
| 2017 | Ganzhou | 0.942244295 | 0.996629495 | 0.964226504 | 0.980507039 |
| 2017 | Guangyuan | 0.999548676 | 1.079463704 | 0.99789109 | 0.927924752 |
| 2017 | Guiyang | 1.019797806 | 1.121231241 | 0.988561957 | 0.920057542 |
| 2017 | Hangzhou | 0.995191436 | 1.167663738 | 1.041205914 | 0.818563189 |
| 2017 | Hefei | 0.964049482 | 1.00729421 | 1.008945726 | 0.948582663 |
| 2017 | Hengyang | 0.977773432 | 1.088234432 | 0.967528238 | 0.928650123 |
| 2017 | Huzhou | 0.923078894 | 0.97036833 | 0.982098444 | 0.968606065 |
| 2017 | Huaihua | 0.890886803 | 1 | 1 | 0.890886803 |
| 2017 | Huai'an | 1.030929773 | 1.129539765 | 0.985747023 | 0.925895734 |
| 2017 | Huaibei | 0.985646476 | 1.128784166 | 0.933142529 | 0.93575527 |
| 2017 | Huainan | 0.993456458 | 1.077356867 | 1.019361226 | 0.904609498 |
| 2017 | Huanggang | 0.705817059 | 1 | 1 | 0.705817059 |
| 2017 | huangshan | 0.98584988 | 1.073105922 | 1.044544407 | 0.879511025 |
| 2017 | Huangshi | 0.986069516 | 1.070280126 | 0.972207805 | 0.94765655 |
| 2017 | Ji'an | 0.843431395 | 0.944000422 | 1.035345196 | 0.862963449 |
| 2017 | Jiaxing | 0.862857233 | 1 | 0.827346345 | 1.04292143 |
| 2017 | Jinhua | 0.776387828 | 0.739980719 | 0.996716554 | 1.052656419 |
| 2017 | Jingmen | 0.96577262 | 1.086887384 | 0.975693885 | 0.910702981 |
| 2017 | Jingzhou | 0.512236932 | 0.513304573 | 1.003355903 | 0.994582341 |
| 2017 | Jingdezhen | 0.915163743 | 0.960104416 | 0.972140516 | 0.980508346 |
| 2017 | Jiujiang | 0.951991069 | 0.986290201 | 0.937348879 | 1.02973836 |
| 2017 | Kunming | 0.898225513 | 1.108614684 | 0.921750474 | 0.879005091 |
| 2017 | Leshan | 0.96072559 | 1.011003548 | 0.992313706 | 0.957629881 |
| 2017 | Lijiang | 0.851440998 | 1 | 1.147570443 | 0.741950966 |
| 2017 | Lianyungang | 0.716517606 | 0.707169246 | 1.000286685 | 1.012929017 |
| 2017 | Liuan | 0.887542582 | 0.978263779 | 0.999088328 | 0.908090933 |
| 2017 | Liupanshui | 0.860507051 | 1 | 1 | 0.860507051 |
| 2017 | Loudi | 0.971472447 | 1.285417224 | 0.842177441 | 0.897393188 |
| 2017 | Luzhou | 1.065478238 | 1.107079197 | 1.000941541 | 0.961517471 |
| 2017 | Meishan | 0.929615473 | 0.907936053 | 0.99635075 | 1.027627766 |
| 2017 | Mianyang | 1.002081261 | 1.122185252 | 0.994522924 | 0.897890933 |
| 2017 | Nanchang | 0.978233875 | 1.068029264 | 0.994720325 | 0.920785674 |
| 2017 | Nanchong | 0.987273278 | 1.135374751 | 0.969210075 | 0.897181337 |
| 2017 | Nanjing | 1.012889728 | 1.235985767 | 1.013921675 | 0.808247351 |
| 2017 | Nantong | 0.904182218 | 1 | 1 | 0.904182218 |
| 2017 | Neijiang | 0.884615383 | 0.877640635 | 0.976572572 | 1.032127244 |
| 2017 | Ningbo | 0.994223169 | 1.071220446 | 1.078824595 | 0.860308447 |
| 2017 | Panzhihua | 1.036983432 | 1 | 1.099803527 | 0.942880621 |
| 2017 | Pingxiang | 0.98379193 | 1.054076052 | 1.011176893 | 0.923005252 |
| 2017 | Qujing | 0.788575635 | 1 | 1 | 0.788575635 |
| 2017 | Quzhou | 0.977264989 | 0.997830852 | 1.001263481 | 0.978153551 |
| 2017 | Shanghai | 1.054764204 | 1 | 1 | 1.054764204 |
| 2017 | Shangrao | 0.871820308 | 0.91006981 | 1.036279312 | 0.924433011 |
| 2017 | Shaoyang | 0.851804983 | 1.023910119 | 0.994788549 | 0.836272015 |
| 2017 | Shaoxing | 0.989980994 | 0.954351685 | 0.949243471 | 1.09280027 |
| 2017 | Shiyan | 0.96730107 | 1.064367271 | 1.005258424 | 0.904049969 |
| 2017 | Suzhou | 0.957845998 | 1 | 1 | 0.957845998 |
| 2017 | Suqian | 0.99968686 | 1.056244767 | 0.996572274 | 0.949709129 |
| 2017 | Suzhou | 0.936851619 | 1.172996011 | 0.943247233 | 0.846737387 |
| 2017 | Suizhou | 0.949457688 | 1 | 0.933095585 | 1.017535291 |
| 2017 | Taizhou | 0.928793477 | 1 | 1.08868739 | 0.853131473 |
| 2017 | Taizhou | 1.182104836 | 1 | 1 | 1.182104836 |
| 2017 | Tongling | 1.054780021 | 1.251300746 | 0.881083119 | 0.956716603 |
| 2017 | Wenzhou | 0.888821749 | 1 | 0.89402277 | 0.994182452 |
| 2017 | Wuxi | 0.963530059 | 1 | 1 | 0.963530059 |
| 2017 | Wuhu | 0.986822621 | 1.031694603 | 1.001748152 | 0.954837326 |
| 2017 | Wuhan | 0.99642198 | 1.075016589 | 1.109588263 | 0.835345772 |
| 2017 | Xianning | 0.923494298 | 0.864633678 | 0.998627819 | 1.069543396 |
| 2017 | Xiangtan | 1.071727497 | 1.203342797 | 0.979472083 | 0.909291117 |
| 2017 | Xiaogan | 0.824012353 | 0.930756622 | 0.9527476 | 0.929222515 |
| 2017 | Xinyu | 1.022420431 | 1 | 1 | 1.022420431 |
| 2017 | Xuzhou | 1.011057351 | 1.187528301 | 1.052068594 | 0.809259425 |
| 2017 | Xuancheng | 0.877005156 | 0.833178025 | 1.095354056 | 0.960969976 |
| 2017 | Yancheng | 0.991206706 | 1 | 1.158966862 | 0.855250256 |
| 2017 | Yangzhou | 0.796096258 | 0.775546768 | 0.960664767 | 1.068527558 |
| 2017 | Yibin | 0.96410723 | 1.031355075 | 0.989746552 | 0.944480797 |
| 2017 | Yichang | 0.920111416 | 0.975640532 | 0.970382569 | 0.971868706 |
| 2017 | Yichun | 0.843356837 | 0.831416563 | 0.995913547 | 1.01852351 |
| 2017 | Yiyang | 0.919290895 | 1.005372916 | 0.958474678 | 0.953992881 |
| 2017 | Yingtan | 0.860353813 | 1 | 1 | 0.860353813 |
| 2017 | yongzhou | 0.862934484 | 0.99011286 | 0.999834926 | 0.871695531 |
| 2017 | Yuxi | 0.725195535 | 0.708415989 | 0.995056945 | 1.028771279 |
| 2017 | Yueyang | 0.955249249 | 1 | 1 | 0.955249249 |
| 2017 | Zhangjiajie | 0.991560903 | 1 | 1 | 0.991560903 |
| 2017 | Changsha | 0.942347597 | 1 | 1 | 0.942347597 |
| 2017 | Zhaotong | 0.832024053 | 1 | 1 | 0.832024053 |
| 2017 | Zhenjiang | 0.959114 | 1 | 1 | 0.959114 |
| 2017 | Chongqing | 1.019263933 | 0.982571031 | 1.180372844 | 0.878827203 |
| 2017 | Zhoushan | 1.137342791 | 1.144860126 | 0.966238286 | 1.028145804 |
| 2017 | Zhuzhou | 0.97722429 | 1.047157926 | 0.976882332 | 0.95530008 |
| 2017 | Ziyang | 0.965494885 | 1 | 1 | 0.965494885 |
| 2017 | Zigong | 0.968220159 | 1 | 1 | 0.968220159 |
| 2017 | Zunyi | 0.641509153 | 0.558124606 | 1.172650034 | 0.9801742 |
| 2018 | Anqing | 0.979888828 | 0.949862502 | 1.053662583 | 0.979071717 |
| 2018 | Anshun | 1.073479599 | 1.082506058 | 0.959168878 | 1.033875826 |
| 2018 | Bazhong | 0.984604321 | 0.981738038 | 0.992368338 | 1.010632405 |
| 2018 | Bengbu | 1.006239781 | 1.02176965 | 1.002474798 | 0.982369841 |
| 2018 | Baoshan | 0.892861107 | 0.960933698 | 0.876399989 | 1.060200778 |
| 2018 | Bozhou | 0.991046417 | 0.989026632 | 0.983169372 | 1.019195902 |
| 2018 | Changde | 1.130054489 | 1 | 1 | 1.130054489 |
| 2018 | Changzhou | 1 | 1 | 1 | 1 |
| 2018 | Chenzhou | 1.063506031 | 1 | 1 | 1.063506031 |
| 2018 | Chengdu | 1.004398987 | 1 | 1.06375931 | 0.9441976 |
| 2018 | Chizhou | 1.005063144 | 1 | 1.006583765 | 0.998489325 |
| 2018 | Chuzhou | 0.945796503 | 0.934301719 | 1.025955378 | 0.986693082 |
| 2018 | Dazhou | 0.983651777 | 0.994180464 | 0.999340211 | 0.990062915 |
| 2018 | Deyang | 1.035983329 | 1 | 1.037583492 | 0.998457798 |
| 2018 | Ezhou | 1.007689031 | 1 | 1.016360261 | 0.991468349 |
| 2018 | Fuzhou | 1.041220582 | 0.921417598 | 1.033093086 | 1.093822334 |
| 2018 | Fuyang | 0.987422001 | 0.987406987 | 0.994352562 | 1.005694804 |
| 2018 | Ganzhou | 1.042705229 | 0.904560228 | 1.064727909 | 1.08264338 |
| 2018 | Guangyuan | 1.033906934 | 1.01821961 | 1.013131221 | 1.002245909 |
| 2018 | Guiyang | 1.015020374 | 1.037651765 | 1.010520327 | 0.968006061 |
| 2018 | Hangzhou | 1.002292715 | 0.998835661 | 1.008613085 | 0.994891995 |
| 2018 | Hefei | 1.001722327 | 1.030793633 | 1.000268963 | 0.971535855 |
| 2018 | Hengyang | 1.034402511 | 0.918318413 | 1.052013516 | 1.070717632 |
| 2018 | Huzhou | 0.991938313 | 0.97846506 | 1.021668597 | 0.992268715 |
| 2018 | Huaihua | 1.031275682 | 1 | 1 | 1.031275682 |
| 2018 | Huai'an | 1.095742624 | 1.007092076 | 1.011578396 | 1.075572851 |
| 2018 | Huaibei | 0.992033201 | 0.977765643 | 1.037089086 | 0.978307472 |
| 2018 | Huainan | 1.015282902 | 1.020288007 | 0.9856071 | 1.009625863 |
| 2018 | Huanggang | 0.993349792 | 1 | 1 | 0.993349792 |
| 2018 | huangshan | 1.003086076 | 1.116828196 | 0.884674823 | 1.015238699 |
| 2018 | Huangshi | 0.991620441 | 0.997087481 | 1.007820834 | 0.986799397 |
| 2018 | Ji'an | 1.04305224 | 0.99151704 | 0.986256991 | 1.066634883 |
| 2018 | Jiaxing | 1.00532012 | 1 | 1.046866685 | 0.960313414 |
| 2018 | Jinhua | 1.121300772 | 1.250578326 | 0.982896361 | 0.912228205 |
| 2018 | Jingmen | 1.02143504 | 1.085748125 | 0.942671655 | 0.997978569 |
| 2018 | Jingzhou | 1.99638446 | 1.98317055 | 0.99959394 | 1.007071954 |
| 2018 | Jingdezhen | 1.040069197 | 1.015904517 | 0.981785312 | 1.042780287 |
| 2018 | Jiujiang | 1.062641789 | 0.923144012 | 1.09342334 | 1.052759317 |
| 2018 | Kunming | 1.134126851 | 1.0680153 | 1.064246812 | 0.997796096 |
| 2018 | Leshan | 1.040378307 | 1.010415798 | 0.996648768 | 1.033115853 |
| 2018 | Lijiang | 0.944825615 | 1 | 0.831373291 | 1.136463758 |
| 2018 | Lianyungang | 1.315016399 | 1.346507195 | 0.990623017 | 0.985857344 |
| 2018 | Liuan | 1.005681154 | 0.985378431 | 0.990141725 | 1.030765556 |
| 2018 | Liupanshui | 1.05225616 | 1 | 0.910076562 | 1.156228172 |
| 2018 | Loudi | 1.031764051 | 0.814691123 | 1.171581757 | 1.080972903 |
| 2018 | Luzhou | 0.935626072 | 0.976486526 | 1.001397411 | 0.956818571 |
| 2018 | Meishan | 1.014865942 | 1.015343265 | 0.966777661 | 1.033877726 |
| 2018 | Mianyang | 0.992373207 | 0.994905251 | 1.00600014 | 0.991505815 |
| 2018 | Nanchang | 0.987115667 | 1.026956671 | 0.986761794 | 0.974100124 |
| 2018 | Nanchong | 1.034540434 | 0.987114977 | 1.013345688 | 1.034241843 |
| 2018 | Nanjing | 0.996867249 | 1.018529706 | 1.005987792 | 0.97290608 |
| 2018 | Nantong | 1.007567802 | 1 | 1 | 1.007567802 |
| 2018 | Neijiang | 0.965493675 | 0.94005736 | 1.047018182 | 0.980936412 |
| 2018 | Ningbo | 1.062020624 | 1.003079938 | 0.996151995 | 1.06284956 |
| 2018 | Panzhihua | 0.995652741 | 1 | 0.988470137 | 1.007266384 |
| 2018 | Pingxiang | 1.029260216 | 1.007076278 | 0.974513174 | 1.048757562 |
| 2018 | Qujing | 0.918591195 | 1 | 0.846346364 | 1.085360835 |
| 2018 | Quzhou | 1.029107647 | 1.016045369 | 0.985480155 | 1.027779194 |
| 2018 | Shanghai | 1.016726288 | 1 | 1 | 1.016726288 |
| 2018 | Shangrao | 1.054766953 | 0.950167102 | 0.996133993 | 1.114393997 |
| 2018 | Shaoyang | 1.033496951 | 0.935696181 | 0.994665999 | 1.110445044 |
| 2018 | Shaoxing | 0.978015264 | 0.882680061 | 1.0436563 | 1.061658442 |
| 2018 | Shiyan | 0.986244532 | 1.001125729 | 1.00069399 | 0.984452336 |
| 2018 | Suzhou | 1.044009164 | 1 | 1 | 1.044009164 |
| 2018 | Suqian | 1.017881365 | 0.976487489 | 1.003053759 | 1.039217067 |
| 2018 | Suzhou | 1.140108846 | 0.902926868 | 1.047363637 | 1.20558059 |
| 2018 | Suizhou | 1.130821597 | 1 | 1.022788606 | 1.105625924 |
| 2018 | Taizhou | 1.003114685 | 0.937232073 | 0.987997073 | 1.083297618 |
| 2018 | Taizhou | 0.975240782 | 1 | 1 | 0.975240782 |
| 2018 | Tongling | 1.00826533 | 1.02178141 | 1.037841682 | 0.950792458 |
| 2018 | Wenzhou | 1.004822614 | 0.864669824 | 1.030876294 | 1.127281929 |
| 2018 | wuxi | 1.086737191 | 1 | 1 | 1.086737191 |
| 2018 | Wuhu | 0.983120619 | 1.043628509 | 0.999472004 | 0.942519267 |
| 2018 | Wuhan | 1.036200017 | 1 | 1.058648522 | 0.978795129 |
| 2018 | Xianning | 1.03178217 | 1.019826722 | 0.974044075 | 1.038682997 |
| 2018 | Xiangtan | 0.994420556 | 1.002081812 | 1.006414526 | 0.986029746 |
| 2018 | Xiaogan | 1.009743788 | 0.967926373 | 1.035927208 | 1.007023551 |
| 2018 | Xinyu | 1.017961393 | 1 | 1 | 1.017961393 |
| 2018 | Xuzhou | 1.097037589 | 1 | 1 | 1.097037589 |
| 2018 | Xuancheng | 0.962170353 | 0.908495892 | 0.99681031 | 1.062469525 |
| 2018 | Yancheng | 1.061656273 | 1 | 1 | 1.061656273 |
| 2018 | Yangzhou | 1.216943867 | 1.289412891 | 1.040945847 | 0.906672413 |
| 2018 | Yibin | 0.974030714 | 0.933089471 | 1.010714009 | 1.03281153 |
| 2018 | Yichang | 0.99878274 | 0.962802222 | 1.027280455 | 1.009822213 |
| 2018 | Yichun | 1.037071509 | 0.934992853 | 0.994296115 | 1.115538773 |
| 2018 | Yiyang | 1.131169066 | 1 | 0.959461504 | 1.17896243 |
| 2018 | Yingtan | 1.00359911 | 1 | 0.793640209 | 1.264551743 |
| 2018 | yongzhou | 1.046236615 | 1.009985872 | 1.000165101 | 1.035721329 |
| 2018 | Yuxi | 1.328068572 | 1.411599985 | 1.00496761 | 0.936174462 |
| 2018 | Yueyang | 1.102992044 | 0.957578148 | 0.972392168 | 1.18455901 |
| 2018 | Zhangjiajie | 1.03236722 | 1 | 0.8917074 | 1.157742125 |
| 2018 | Changsha | 1.061179551 | 1 | 1 | 1.061179551 |
| 2018 | Zhaotong | 0.957383034 | 1 | 1 | 0.957383034 |
| 2018 | Zhenjiang | 1.078496909 | 1 | 1 | 1.078496909 |
| 2018 | Chongqing | 1.00708283 | 0.954423156 | 1.036837477 | 1.017685384 |
| 2018 | Zhoushan | 1.280699315 | 1.163460879 | 1.038239461 | 1.060224562 |
| 2018 | Zhuzhou | 1.023796473 | 0.990507431 | 1.017278763 | 1.016051949 |
| 2018 | Ziyang | 0.968068737 | 1 | 1 | 0.968068737 |
| 2018 | Zigong | 1.129841257 | 1 | 1 | 1.129841257 |
| 2018 | Zunyi | 1.193236965 | 1.013144033 | 1.039583027 | 1.132912393 |
| 2019 | Anqing | 1.085384278 | 1.10510383 | 1.013067334 | 0.969487313 |
| 2019 | Anshun | 0.990947587 | 1.055769353 | 0.959211142 | 0.978514844 |
| 2019 | Bazhong | 1.058795655 | 1.268131586 | 0.983885562 | 0.848600421 |
| 2019 | Bengbu | 1.065401814 | 1.124991234 | 0.988262874 | 0.95827865 |
| 2019 | Baoshan | 1.251350178 | 1.040654524 | 1.019026226 | 1.180013353 |
| 2019 | Bozhou | 1.150475437 | 1.217854242 | 0.986529173 | 0.957573469 |
| 2019 | Changde | 0.949032367 | 1 | 1 | 0.949032367 |
| 2019 | Changzhou | 1 | 1 | 1 | 1 |
| 2019 | chenzhou | 0.957322525 | 0.955630614 | 0.999807033 | 1.001963812 |
| 2019 | Chengdu | 1.003742757 | 1 | 1.053819733 | 0.95248051 |
| 2019 | Chizhou | 1.064800375 | 1 | 1.039484503 | 1.024354256 |
| 2019 | Chuzhou | 1.232418099 | 1.247905752 | 1.00533779 | 0.98234553 |
| 2019 | Dazhou | 1.101577778 | 1.216245032 | 0.989212993 | 0.915596819 |
| 2019 | Deyang | 1.055979796 | 0.9971054 | 0.98710951 | 1.072875196 |
| 2019 | Ezhou | 1.026625333 | 1 | 1.03491593 | 0.991989111 |
| 2019 | Fuzhou | 1.005071303 | 1.027965366 | 0.985892819 | 0.991719122 |
| 2019 | Fuyang | 1.160824367 | 1.314663522 | 0.999180174 | 0.883706593 |
| 2019 | Ganzhou | 1.056025018 | 1.057320237 | 1.016313662 | 0.982742863 |
| 2019 | Guangyuan | 1.053612983 | 1.15166424 | 0.966580508 | 0.946492547 |
| 2019 | Guiyang | 0.971260569 | 0.987302761 | 0.995519334 | 0.988179199 |
| 2019 | Hangzhou | 1.149521833 | 1.006407254 | 1.023483474 | 1.115995983 |
| 2019 | Hefei | 1.037517155 | 1.086721063 | 1.001881862 | 0.952929316 |
| 2019 | Hengyang | 1.001919545 | 1.020278945 | 1.006606413 | 0.975560553 |
| 2019 | Huzhou | 1.01785999 | 1.030185789 | 1.000635004 | 0.987408355 |
| 2019 | Huaihua | 0.979595574 | 0.943178417 | 0.97028865 | 1.070414562 |
| 2019 | Huai'an | 1.037029158 | 1.024023073 | 1.001159781 | 1.011527818 |
| 2019 | Huaibei | 1.029132335 | 0.989530917 | 1.026531238 | 1.013140523 |
| 2019 | Huainan | 1.058116343 | 1.07784129 | 0.976689075 | 1.005130094 |
| 2019 | Huanggang | 1.017529828 | 0.927859582 | 0.990005491 | 1.107713091 |
| 2019 | huangshan | 1.044115239 | 1 | 1.007339328 | 1.036507967 |
| 2019 | Huangshi | 1.019253022 | 1.037893483 | 1.002689586 | 0.979405906 |
| 2019 | Ji'an | 1.021352877 | 1.048901809 | 1.012354697 | 0.96185206 |
| 2019 | Jiaxing | 1.041420638 | 0.823478794 | 1.109103623 | 1.140254083 |
| 2019 | Jinhua | 1.064905252 | 0.94191906 | 0.985155089 | 1.147605918 |
| 2019 | Jingmen | 1.003564625 | 0.999675284 | 1.020614484 | 0.983613911 |
| 2019 | Jingzhou | 1.058782011 | 1.074055992 | 0.992619485 | 0.993108812 |
| 2019 | Jingdezhen | 1.082160846 | 1.082878361 | 1.013514803 | 0.986011647 |
| 2019 | Jiujiang | 1.034618545 | 1.048146747 | 1.002060514 | 0.985063481 |
| 2019 | Kunming | 1.064043991 | 1.071385872 | 1.020822969 | 0.972888871 |
| 2019 | Leshan | 1.041216492 | 1.045672336 | 1.004868312 | 0.990914694 |
| 2019 | Lijiang | 1.131198863 | 1 | 0.871217436 | 1.298411644 |
| 2019 | Lianyungang | 1.069297087 | 1.051663198 | 1.007324451 | 1.009374506 |
| 2019 | Liuan | 1.070384654 | 1.097509343 | 0.994979178 | 0.980206674 |
| 2019 | Liupanshui | 0.880350498 | 0.833022041 | 1.046708112 | 1.009656235 |
| 2019 | Loudi | 1.003191971 | 0.976028035 | 1.0237041 | 1.004031441 |
| 2019 | Luzhou | 1.032449026 | 1.099775513 | 0.999412238 | 0.939333714 |
| 2019 | Meishan | 0.959359302 | 1.02850199 | 0.973983813 | 0.957688816 |
| 2019 | Mianyang | 1.092603472 | 1.136103991 | 1.003002233 | 0.958832158 |
| 2019 | Nanchang | 0.982954487 | 1.012078443 | 1.017576328 | 0.954447928 |
| 2019 | Nanchong | 1.050929468 | 1.145397595 | 1.014981294 | 0.903980921 |
| 2019 | Nanjing | 1.093058659 | 1 | 1.005226581 | 1.087375404 |
| 2019 | Nantong | 1.341883796 | 1 | 1 | 1.341883796 |
| 2019 | Neijiang | 0.967391976 | 1.029690926 | 0.971454477 | 0.967103915 |
| 2019 | Ningbo | 1.073315799 | 1.047072112 | 0.984987778 | 1.0406869 |
| 2019 | Panzhihua | 0.925675691 | 0.781706368 | 1.201138283 | 0.985875786 |
| 2019 | Pingxiang | 0.949721285 | 1.011774114 | 0.952682674 | 0.985290603 |
| 2019 | Qujing | 1.217644 | 1 | 1.003172949 | 1.213792698 |
| 2019 | Quzhou | 0.990662558 | 1.014042699 | 1.005081192 | 0.972004691 |
| 2019 | Shanghai | 1.112113044 | 1 | 1 | 1.112113044 |
| 2019 | Shangrao | 1.021685456 | 0.96336597 | 1.009803125 | 1.050241559 |
| 2019 | Shaoyang | 1.045534853 | 1.068722969 | 1.010892054 | 0.967762051 |
| 2019 | Shaoxing | 1.001653412 | 0.973192263 | 1.001873909 | 1.027320041 |
| 2019 | Shiyan | 1.02528071 | 1.075398498 | 0.984849586 | 0.968062627 |
| 2019 | Suzhou | 1 | 1 | 1 | 1 |
| 2019 | Suqian | 1.051972077 | 1.020937674 | 0.99452949 | 1.036065748 |
| 2019 | Suzhou | 1.093222125 | 1.076500612 | 0.973278039 | 1.043415316 |
| 2019 | Suizhou | 1.091198579 | 1 | 1.124837739 | 0.970094211 |
| 2019 | Taizhou | 0.989494083 | 0.96133509 | 1.006516072 | 1.02262803 |
| 2019 | Taizhou | 0.745537256 | 0.899276858 | 0.991783516 | 0.835909089 |
| 2019 | Tongling | 0.869516132 | 0.766675062 | 1.142907855 | 0.992327642 |
| 2019 | Wenzhou | 1.043753344 | 0.974604242 | 1.032276848 | 1.03746486 |
| 2019 | Wuxi | 1 | 1 | 1 | 1 |
| 2019 | Wuhu | 1.068956583 | 1.041300038 | 1.001629007 | 1.024890078 |
| 2019 | Wuhan | 1.089774788 | 1 | 1 | 1.089774788 |
| 2019 | Xianning | 1.039756043 | 1.091749799 | 0.990187275 | 0.961813773 |
| 2019 | Xiangtan | 1.003835725 | 1.037997907 | 0.98176172 | 0.985054081 |
| 2019 | Xiaogan | 1.055771895 | 1.078167388 | 0.997632042 | 0.981552461 |
| 2019 | Xinyu | 0.920423236 | 1 | 0.750175707 | 1.226943539 |
| 2019 | Xuzhou | 1.090596677 | 1 | 1 | 1.090596677 |
| 2019 | Xuancheng | 1.048835373 | 1.094753215 | 0.97374689 | 0.983886526 |
| 2019 | Yancheng | 1.205238441 | 1 | 0.94629088 | 1.273644781 |
| 2019 | Yangzhou | 1.089834884 | 1 | 1 | 1.089834884 |
| 2019 | Yibin | 1.094508625 | 1.1762976 | 0.994806664 | 0.935326613 |
| 2019 | Yichang | 0.993685731 | 1.007571056 | 1.004232913 | 0.982062028 |
| 2019 | Yichun | 1.077024543 | 1.077342416 | 0.998010082 | 1.001698245 |
| 2019 | Yiyang | 0.913035348 | 0.90735956 | 1.057544819 | 0.95150131 |
| 2019 | Yingtan | 1.069724894 | 1 | 1.079724388 | 0.990738846 |
| 2019 | Yongzhou | 1.029257716 | 1 | 1 | 1.029257716 |
| 2019 | Yuxi | 1.494389177 | 1 | 1 | 1.494389177 |
| 2019 | Yueyang | 0.913904474 | 1.04430119 | 1.016433955 | 0.860985556 |
| 2019 | Zhangjiajie | 0.892756924 | 1 | 0.95382184 | 0.935978698 |
| 2019 | Changsha | 0.895554518 | 1 | 1 | 0.895554518 |
| 2019 | Zhaotong | 1.072315425 | 1 | 1 | 1.072315425 |
| 2019 | Zhenjiang | 0.985657592 | 0.999991371 | 0.999075498 | 0.98657819 |
| 2019 | Chongqing | 1.065996004 | 1.08599506 | 0.998857852 | 0.982706976 |
| 2019 | Zhoushan | 0.778684918 | 0.901841535 | 0.95113501 | 0.907798295 |
| 2019 | Zhuzhou | 1.042849503 | 1.136587139 | 1.007504737 | 0.910692593 |
| 2019 | Ziyang | 0.906436505 | 1 | 0.967309302 | 0.937069977 |
| 2019 | Zigong | 0.974106434 | 1 | 1 | 0.974106434 |
| 2019 | Zunyi | 1.292539471 | 1.422447552 | 0.999988194 | 0.908683578 |
