# Supplementary material for: Research on coupling coordination and influencing factors between Urban low-carbon economy efficiency and digital finance—Evidence from 100 cities in China’s Yangtze River economic belt
Source: PLoS One. 2022 Jul 29;17(7):e0271455. doi: 10.1371/journal.pone.0271455 (PMC9337701; doi:10.1371/journal.pone.0271455)
Supplement: S2 Table — (DOCX) [file pone.0271455.s002.docx]

**S2 Table. Coupling coordination degree of urban low carbon economy efficiency and digital finance from 2012 to 2019**

| **Period** | **City** | **Coupling degree** | **coordination degree** | **Coupling coordination degree** |
| --- | --- | --- | --- | --- |
| 2012 | Anqing | 0.809085317 | 0.216835868 | 0.418854052 |
| 2012 | Anshun | 0.470511336 | 0.206670399 | 0.311834516 |
| 2012 | Bazhong | 0.517648424 | 0.200856825 | 0.322448784 |
| 2012 | Bengbu | 0.888237635 | 0.223513841 | 0.445570875 |
| 2012 | Baoshan | 0.515038674 | 0.222216885 | 0.338305025 |
| 2012 | Bozhou | 0.546991568 | 0.185664462 | 0.318679926 |
| 2012 | Changde | 0.980947279 | 0.278380021 | 0.522566861 |
| 2012 | Changzhou | 0.790243503 | 0.215970859 | 0.413121735 |
| 2012 | Chenzhou | 0.985226191 | 0.282501012 | 0.527567432 |
| 2012 | Chengdu | 0.861069373 | 0.230561443 | 0.445566378 |
| 2012 | Chizhou | 0.80738297 | 0.222017822 | 0.423383288 |
| 2012 | Chuzhou | 0.593715938 | 0.186162453 | 0.332456938 |
| 2012 | Dazhou | 0.903035308 | 0.228711829 | 0.454461063 |
| 2012 | Deyang | 0.876123356 | 0.236063414 | 0.454775407 |
| 2012 | Ezhou | 0.760922918 | 0.212912866 | 0.402505005 |
| 2012 | Fuzhou | 0.786694609 | 0.197802033 | 0.394474071 |
| 2012 | Fuyang | 0.841971788 | 0.221172297 | 0.431533121 |
| 2012 | Ganzhou | 0.826956117 | 0.192801826 | 0.399297695 |
| 2012 | Guangyuan | 0.92643105 | 0.280770372 | 0.510014108 |
| 2012 | Guiyang | 0.999433333 | 0.342486143 | 0.58505732 |
| 2012 | Hangzhou | 0.965031528 | 0.274695682 | 0.51486891 |
| 2012 | Hefei | 0.792454795 | 0.200956089 | 0.399059665 |
| 2012 | Hengyang | 0.979972513 | 0.276750194 | 0.520775944 |
| 2012 | Huzhou | 0.726636615 | 0.193471641 | 0.374944767 |
| 2012 | Huaihua | 0.86963019 | 0.248707419 | 0.465062877 |
| 2012 | Huai'an | 0.817590963 | 0.213390572 | 0.417691517 |
| 2012 | Huaibei | 0.915949626 | 0.24145878 | 0.470280852 |
| 2012 | Huainan | 0.802395841 | 0.195172224 | 0.395733977 |
| 2012 | Huanggang | 0.941564053 | 0.243023346 | 0.478353475 |
| 2012 | huangshan | 0.832359794 | 0.220695846 | 0.428600454 |
| 2012 | Huangshi | 0.705853964 | 0.206568618 | 0.381847192 |
| 2012 | Ji'an | 0.988673415 | 0.2996642 | 0.544306925 |
| 2012 | Jiaxing | 0.988844426 | 0.287090779 | 0.53281152 |
| 2012 | Jinhua | 0.862635366 | 0.218251365 | 0.433902461 |
| 2012 | Jingmen | 0.866387857 | 0.233134101 | 0.449426917 |
| 2012 | Jingzhou | 0.922577011 | 0.224523074 | 0.455126165 |
| 2012 | Jingdezhen | 0.876564475 | 0.214682877 | 0.433801087 |
| 2012 | Jiujiang | 0.984030835 | 0.243486503 | 0.489487719 |
| 2012 | Kunming | 0.912412247 | 0.195619692 | 0.422475801 |
| 2012 | Leshan | 0.858382754 | 0.230725069 | 0.445028561 |
| 2012 | Lijiang | 0.93416161 | 0.228546635 | 0.462060052 |
| 2012 | Lianyungang | 0.733374419 | 0.202356467 | 0.385231172 |
| 2012 | Liuan | 0.491406903 | 0.172198536 | 0.290894395 |
| 2012 | Liupanshui | 0.804725925 | 0.190438403 | 0.391472502 |
| 2012 | Loudi | 0.741723887 | 0.195582588 | 0.380878298 |
| 2012 | Luzhou | 0.836355216 | 0.214672509 | 0.423724525 |
| 2012 | Meishan | 0.883424027 | 0.242238168 | 0.462600279 |
| 2012 | Mianyang | 0.969080778 | 0.261668363 | 0.503565071 |
| 2012 | Nanchang | 0.994187219 | 0.298240005 | 0.544524013 |
| 2012 | Nanchong | 0.958702349 | 0.260423992 | 0.499668983 |
| 2012 | Nanjing | 0.743216242 | 0.204731052 | 0.390076202 |
| 2012 | Nantong | 0.992855784 | 0.293614409 | 0.539922925 |
| 2012 | Neijiang | 0.785264256 | 0.204815077 | 0.401041094 |
| 2012 | Ningbo | 0.709649021 | 0.193219379 | 0.370294401 |
| 2012 | Panzhihua | 0.868233013 | 0.235239669 | 0.451932347 |
| 2012 | Pingxiang | 0.000108684 | 0.169317406 | 0.004289759 |
| 2012 | Qujing | 0.813952491 | 0.414267692 | 0.580684269 |
| 2012 | Quzhou | 0.998041377 | 0.299914314 | 0.547107754 |
| 2012 | Shanghai | 0.801978919 | 0.213620104 | 0.413906777 |
| 2012 | Shangrao | 0.61766249 | 0.200932669 | 0.352290466 |
| 2012 | Shaoyang | 0.992405248 | 0.249654123 | 0.497753013 |
| 2012 | Shaoxing | 0.876864291 | 0.221949219 | 0.441156826 |
| 2012 | Shiyan | 0.983890991 | 0.291979825 | 0.535981641 |
| 2012 | Suzhou | 0.856835689 | 0.21684541 | 0.431046269 |
| 2012 | Suqian | 0.718485053 | 0.201036845 | 0.380055217 |
| 2012 | Suzhou | 0.779180683 | 0.187642023 | 0.382370291 |
| 2012 | Suizhou | 0.981231577 | 0.299548639 | 0.542149964 |
| 2012 | Taizhou | 0.945485096 | 0.247737952 | 0.483975765 |
| 2012 | Taizhou | 0.968122051 | 0.262818215 | 0.504420568 |
| 2012 | Tongling | 0.997608529 | 0.269793382 | 0.518794929 |
| 2012 | Wenzhou | 0.975146033 | 0.280271168 | 0.52278611 |
| 2012 | wuxi | 0.922598267 | 0.265956299 | 0.495349191 |
| 2012 | Wuhu | 0.988957844 | 0.272727418 | 0.519341813 |
| 2012 | Wuhan | 0.774305694 | 0.214737371 | 0.407765091 |
| 2012 | Xianning | 0.950140729 | 0.219749413 | 0.456938582 |
| 2012 | Xiangtan | 0.813704489 | 0.219310714 | 0.422438294 |
| 2012 | Xiaogan | 0.967803284 | 0.158804395 | 0.392034967 |
| 2012 | Xinyu | 0.924200296 | 0.24678612 | 0.477577015 |
| 2012 | Xuzhou | 0.882610677 | 0.22287463 | 0.443521733 |
| 2012 | Xuancheng | 0.9325767 | 0.213504568 | 0.446216747 |
| 2012 | Yancheng | 0.938691899 | 0.257131389 | 0.491291311 |
| 2012 | Yangzhou | 0.837663268 | 0.201758036 | 0.411102537 |
| 2012 | Yibin | 0.928809311 | 0.243804785 | 0.475865689 |
| 2012 | Yichang | 0.781610347 | 0.208551214 | 0.403739751 |
| 2012 | Yichun | 0.79038473 | 0.198667371 | 0.396262106 |
| 2012 | Yiyang | 0.84207487 | 0.21047512 | 0.420993835 |
| 2012 | Yingtan | 0.742455114 | 0.187168137 | 0.372778675 |
| 2012 | Yongzhou | 0.866093446 | 0.2158884 | 0.432411295 |
| 2012 | Yuxi | 0.84428974 | 0.219440237 | 0.430431343 |
| 2012 | yueyang | 0.824271738 | 0.225803044 | 0.431419827 |
| 2012 | Zhangjiajie | 0.986054406 | 0.280862465 | 0.526256279 |
| 2012 | Changsha | 0.815938631 | 0.205260329 | 0.409242998 |
| 2012 | Zhaotong | 0.172031493 | 0.116266794 | 0.141426837 |
| 2012 | Zhenjiang | 0.955123024 | 0.265063294 | 0.503158081 |
| 2012 | Chongqing | 0.938268098 | 0.218127956 | 0.452396399 |
| 2012 | Zhoushan | 0.98042482 | 0.281062707 | 0.524938905 |
| 2012 | Zhuzhou | 0.899569765 | 0.226784204 | 0.451672684 |
| 2012 | Ziyang | 0.700323498 | 0.228552249 | 0.400075631 |
| 2012 | Zigong | 0.851370697 | 0.225962051 | 0.43860856 |
| 2012 | Zunyi | 0.769439941 | 0.198524148 | 0.390835526 |
| 2013 | Anqing | 0.996138187 | 0.266451105 | 0.515191344 |
| 2013 | Anshun | 0.977335346 | 0.242852062 | 0.487183645 |
| 2013 | bazhong | 0.980538097 | 0.185486028 | 0.426469362 |
| 2013 | Bengbu | 0.996549651 | 0.297442657 | 0.544441343 |
| 2013 | Baoshan | 0.92370136 | 0.25101474 | 0.481521191 |
| 2013 | Bozhou | 0.957072105 | 0.227812601 | 0.466940131 |
| 2013 | Changde | 0.9812321 | 0.322794502 | 0.562793325 |
| 2013 | Changzhou | 0.989696111 | 0.275267657 | 0.521949547 |
| 2013 | chenzhou | 0.995106772 | 0.34750647 | 0.588052754 |
| 2013 | Chengdu | 0.921178196 | 0.413910258 | 0.617482878 |
| 2013 | Chizhou | 0.997680702 | 0.263702904 | 0.512924262 |
| 2013 | Chuzhou | 0.94719597 | 0.245173795 | 0.481900021 |
| 2013 | Dazhou | 0.98898721 | 0.297349589 | 0.54228677 |
| 2013 | Deyang | 0.995360419 | 0.267860161 | 0.516350077 |
| 2013 | Ezhou | 0.982086369 | 0.284349603 | 0.528446657 |
| 2013 | Fuzhou | 0.974532675 | 0.271706729 | 0.514574665 |
| 2013 | Fuyang | 0.98715684 | 0.293302941 | 0.538085499 |
| 2013 | Ganzhou | 0.96202307 | 0.263848415 | 0.503813718 |
| 2013 | Guangyuan | 0.999718272 | 0.30680848 | 0.553824922 |
| 2013 | Guiyang | 0.999854467 | 0.481860723 | 0.694111372 |
| 2013 | Hangzhou | 0.999086017 | 0.343470967 | 0.585796074 |
| 2013 | Hefei | 0.983872345 | 0.282868806 | 0.527547908 |
| 2013 | Hengyang | 0.996598952 | 0.351846684 | 0.592157104 |
| 2013 | Huzhou | 0.959868289 | 0.266271143 | 0.505554375 |
| 2013 | Huaihua | 0.993695654 | 0.296089302 | 0.542422947 |
| 2013 | Huai'an | 0.986347654 | 0.26989966 | 0.51596017 |
| 2013 | Huaibei | 0.994695621 | 0.302568473 | 0.548601435 |
| 2013 | Huainan | 0.985112866 | 0.270210238 | 0.515933699 |
| 2013 | Huanggang | 0.99911307 | 0.320454079 | 0.56583554 |
| 2013 | huangshan | 0.996602201 | 0.300511534 | 0.547257212 |
| 2013 | Huangshi | 0.973339368 | 0.274313147 | 0.51672022 |
| 2013 | Ji'an | 0.995620637 | 0.365788642 | 0.603478849 |
| 2013 | Jiaxing | 0.994991118 | 0.367276413 | 0.604513663 |
| 2013 | Jinhua | 0.992088305 | 0.261741314 | 0.509578744 |
| 2013 | Jingmen | 0.994281849 | 0.307822536 | 0.553229031 |
| 2013 | Jingzhou | 0.997849937 | 0.319668401 | 0.564784113 |
| 2013 | Jingdezhen | 0.988362287 | 0.29329649 | 0.538408014 |
| 2013 | Jiujiang | 0.998913573 | 0.338893044 | 0.581828893 |
| 2013 | Kunming | 0.93467229 | 0.39589777 | 0.608304755 |
| 2013 | Leshan | 0.999804761 | 0.279000064 | 0.528153001 |
| 2013 | Lijiang | 0.942602239 | 0.427592545 | 0.634861946 |
| 2013 | Lianyungang | 0.982863565 | 0.261387083 | 0.50686077 |
| 2013 | Liuan | 0.967901602 | 0.246138188 | 0.488095838 |
| 2013 | Liupanshui | 0.967981533 | 0.279877329 | 0.520496 |
| 2013 | Loudi | 0.985813205 | 0.253014013 | 0.499424223 |
| 2013 | Luzhou | 0.964458807 | 0.279824756 | 0.51949923 |
| 2013 | Meishan | 0.999425166 | 0.294447485 | 0.542474171 |
| 2013 | Mianyang | 0.998987586 | 0.363568895 | 0.602661441 |
| 2013 | Nanchang | 0.985094466 | 0.364943098 | 0.599586045 |
| 2013 | Nanchong | 0.990556166 | 0.394303058 | 0.62496346 |
| 2013 | Nanjing | 0.981790654 | 0.262538576 | 0.507698651 |
| 2013 | Nantong | 0.913751016 | 0.290344968 | 0.515075732 |
| 2013 | Neijiang | 0.991127833 | 0.24570789 | 0.493485489 |
| 2013 | Ningbo | 0.969341004 | 0.264530285 | 0.506379355 |
| 2013 | Panzhihua | 0.99999571 | 0.258528585 | 0.508455972 |
| 2013 | Pingxiang | 0.941800446 | 0.226533859 | 0.461897921 |
| 2013 | Qujing | 0.999934246 | 0.332461141 | 0.576575477 |
| 2013 | Quzhou | 0.983856558 | 0.39203513 | 0.621052601 |
| 2013 | Shanghai | 0.975582613 | 0.282638237 | 0.525106609 |
| 2013 | Shangrao | 0.961445273 | 0.248116282 | 0.488416039 |
| 2013 | Shaoyang | 0.993724905 | 0.325760284 | 0.56896055 |
| 2013 | Shaoxing | 0.987196904 | 0.232941765 | 0.479540811 |
| 2013 | Shiyan | 0.997435143 | 0.373195672 | 0.610113497 |
| 2013 | Suzhou | 0.99886797 | 0.248493064 | 0.498208553 |
| 2013 | Suqian | 0.989339914 | 0.224927922 | 0.471731037 |
| 2013 | Suzhou | 0.986638373 | 0.274028819 | 0.519968604 |
| 2013 | Suizhou | 0.994998021 | 0.345901585 | 0.586661224 |
| 2013 | Taizhou | 0.999985504 | 0.321402402 | 0.566919521 |
| 2013 | Taizhou | 0.961375946 | 0.24200708 | 0.4823482 |
| 2013 | Tongling | 0.993286249 | 0.371630776 | 0.60756542 |
| 2013 | Wenzhou | 0.998089587 | 0.361799956 | 0.600923264 |
| 2013 | wuxi | 0.997956151 | 0.283562934 | 0.531961816 |
| 2013 | Wuhu | 0.994495349 | 0.37083509 | 0.60728393 |
| 2013 | Wuhan | 0.982821307 | 0.271095702 | 0.516176938 |
| 2013 | Xianning | 0.995936673 | 0.312068121 | 0.557494471 |
| 2013 | Xiangtan | 0.990224974 | 0.276365566 | 0.523129129 |
| 2013 | Xiaogan | 0.99458417 | 0.294161104 | 0.540895533 |
| 2013 | Xinyu | 0.999465605 | 0.300648443 | 0.548167655 |
| 2013 | Xuzhou | 0.999283798 | 0.299936425 | 0.547468364 |
| 2013 | Xuancheng | 0.997615611 | 0.297443485 | 0.544733205 |
| 2013 | Yancheng | 0.997255401 | 0.303804016 | 0.550427285 |
| 2013 | Yangzhou | 0.998630401 | 0.233469974 | 0.482856308 |
| 2013 | Yibin | 0.988160385 | 0.359269201 | 0.595831849 |
| 2013 | Yichang | 0.98273498 | 0.274320585 | 0.519215211 |
| 2013 | Yichun | 0.980752195 | 0.264934657 | 0.509740372 |
| 2013 | Yiyang | 0.994088603 | 0.287827097 | 0.534907129 |
| 2013 | Yingtan | 0.964598085 | 0.268069416 | 0.508506878 |
| 2013 | yongzhou | 0.985390837 | 0.280041981 | 0.525310196 |
| 2013 | Yuxi | 0.978264616 | 0.298721194 | 0.540581515 |
| 2013 | yueyang | 0.990597383 | 0.27573386 | 0.522629161 |
| 2013 | Zhangjiajie | 0.998644991 | 0.368519121 | 0.606646334 |
| 2013 | Changsha | 0.973954292 | 0.283892673 | 0.525831235 |
| 2013 | Zhaotong | 0.862409975 | 0.287847901 | 0.498239803 |
| 2013 | Zhenjiang | 0.99817607 | 0.315239631 | 0.56094978 |
| 2013 | Chongqing | 0.998855523 | 0.287839104 | 0.53619929 |
| 2013 | Zhoushan | 0.999054854 | 0.352424988 | 0.593373318 |
| 2013 | Zhuzhou | 0.997594698 | 0.304824212 | 0.551444483 |
| 2013 | Ziyang | 0.992914397 | 0.222416437 | 0.469936679 |
| 2013 | Zigong | 0.974959513 | 0.307414461 | 0.547463837 |
| 2013 | Zunyi | 0.869864745 | 0.383361361 | 0.577470807 |
| 2014 | Anqing | 0.999773202 | 0.328713828 | 0.573270684 |
| 2014 | Anshun | 0.995160452 | 0.324946789 | 0.568659998 |
| 2014 | bazhong | 0.988703757 | 0.24784899 | 0.495024471 |
| 2014 | Bengbu | 0.999397583 | 0.333559315 | 0.577371954 |
| 2014 | Baoshan | 0.995103018 | 0.303997478 | 0.550008007 |
| 2014 | Bozhou | 0.98290821 | 0.297001997 | 0.540301491 |
| 2014 | Changde | 0.987704986 | 0.378987424 | 0.611823314 |
| 2014 | Changzhou | 0.97870941 | 0.308057916 | 0.549089411 |
| 2014 | chenzhou | 0.995741574 | 0.392003961 | 0.62476767 |
| 2014 | Chengdu | 0.943043449 | 0.231115399 | 0.466853149 |
| 2014 | Chizhou | 0.999979603 | 0.348863522 | 0.590640675 |
| 2014 | Chuzhou | 0.978332787 | 0.292042109 | 0.534522563 |
| 2014 | Dazhou | 0.99906761 | 0.315082281 | 0.561060159 |
| 2014 | Deyang | 0.990183752 | 0.388673202 | 0.620369156 |
| 2014 | Ezhou | 0.996729712 | 0.312029194 | 0.557681601 |
| 2014 | Fuzhou | 0.987284533 | 0.298385645 | 0.542762869 |
| 2014 | Fuyang | 0.996395565 | 0.32973092 | 0.573186206 |
| 2014 | Ganzhou | 0.999227916 | 0.274206731 | 0.523445337 |
| 2014 | Guangyuan | 0.999519305 | 0.363522819 | 0.602783606 |
| 2014 | Guiyang | 0.947890589 | 0.401261138 | 0.616726566 |
| 2014 | Hangzhou | 0.992066287 | 0.377112383 | 0.61165389 |
| 2014 | Hefei | 0.996095497 | 0.304954513 | 0.551147727 |
| 2014 | Hengyang | 0.992595236 | 0.380086119 | 0.614224447 |
| 2014 | Huzhou | 0.992793666 | 0.277943959 | 0.525300868 |
| 2014 | Huaihua | 0.999042333 | 0.343160741 | 0.585518665 |
| 2014 | Huai'an | 0.999846378 | 0.331334284 | 0.575572223 |
| 2014 | Huaibei | 0.999827345 | 0.34134528 | 0.58419718 |
| 2014 | Huainan | 0.996658374 | 0.319079789 | 0.563926896 |
| 2014 | Huanggang | 0.999930427 | 0.358802688 | 0.598980571 |
| 2014 | huangshan | 0.999351986 | 0.344634061 | 0.586865175 |
| 2014 | Huangshi | 0.99304853 | 0.299494737 | 0.545355671 |
| 2014 | Ji'an | 0.984297464 | 0.37117461 | 0.60443877 |
| 2014 | Jiaxing | 0.989517407 | 0.395117856 | 0.625280734 |
| 2014 | Jinhua | 0.996239705 | 0.335445579 | 0.578086676 |
| 2014 | Jingmen | 0.999976938 | 0.322910016 | 0.568245166 |
| 2014 | Jingzhou | 0.999999719 | 0.339995103 | 0.583090908 |
| 2014 | Jingdezhen | 0.999998706 | 0.323427777 | 0.568706742 |
| 2014 | Jiujiang | 0.994538216 | 0.372227619 | 0.608436186 |
| 2014 | Kunming | 0.997532222 | 0.273937343 | 0.522744036 |
| 2014 | Leshan | 0.990891416 | 0.340930241 | 0.58122702 |
| 2014 | Lijiang | 0.997291464 | 0.313270808 | 0.558947496 |
| 2014 | Lianyungang | 0.998246158 | 0.297492856 | 0.544950549 |
| 2014 | Liuan | 0.927145144 | 0.369486245 | 0.585292558 |
| 2014 | Liupanshui | 0.973711486 | 0.328121086 | 0.565239127 |
| 2014 | Loudi | 0.996189316 | 0.312025867 | 0.55752743 |
| 2014 | Luzhou | 0.999917048 | 0.280606646 | 0.529701207 |
| 2014 | Meishan | 0.994818798 | 0.370727659 | 0.607294693 |
| 2014 | Mianyang | 0.976506844 | 0.34222523 | 0.578087605 |
| 2014 | Nanchang | 0.990449698 | 0.421118744 | 0.645830421 |
| 2014 | Nanchong | 0.954545543 | 0.304667178 | 0.539276086 |
| 2014 | Nanjing | 0.98619338 | 0.291617778 | 0.536275603 |
| 2014 | Nantong | 0.979845334 | 0.398305982 | 0.624722545 |
| 2014 | Neijiang | 0.987908347 | 0.33141819 | 0.572198214 |
| 2014 | Ningbo | 0.999744549 | 0.343155093 | 0.585719586 |
| 2014 | Panzhihua | 0.987631112 | 0.363766176 | 0.599388683 |
| 2014 | Pingxiang | 0.977189535 | 0.296903995 | 0.53863854 |
| 2014 | Qujing | 0.996323156 | 0.359443923 | 0.598433207 |
| 2014 | Quzhou | 0.977858318 | 0.415846342 | 0.637682369 |
| 2014 | Shanghai | 0.995509253 | 0.329569051 | 0.572790573 |
| 2014 | Shangrao | 0.970994587 | 0.297615329 | 0.537571273 |
| 2014 | Shaoyang | 0.99352617 | 0.390795519 | 0.623109601 |
| 2014 | Shaoxing | 0.999702454 | 0.322610692 | 0.567903777 |
| 2014 | Shiyan | 0.986256444 | 0.393582589 | 0.623035604 |
| 2014 | Suzhou | 0.996311825 | 0.319339279 | 0.564058064 |
| 2014 | Suqian | 0.995543287 | 0.313702409 | 0.558841952 |
| 2014 | Suzhou | 0.996668578 | 0.312451944 | 0.558042144 |
| 2014 | Suizhou | 0.996122961 | 0.392390293 | 0.625195154 |
| 2014 | Taizhou | 0.996573045 | 0.355480593 | 0.595199443 |
| 2014 | Taizhou | 0.995773058 | 0.381472019 | 0.616327477 |
| 2014 | Tongling | 0.987933814 | 0.396707958 | 0.626036106 |
| 2014 | Wenzhou | 0.988855094 | 0.390765081 | 0.621618887 |
| 2014 | wuxi | 0.997731606 | 0.350576709 | 0.591423252 |
| 2014 | Wuhu | 0.98738898 | 0.397326811 | 0.626351431 |
| 2014 | Wuhan | 0.99744109 | 0.327816267 | 0.57181939 |
| 2014 | Xianning | 0.999671437 | 0.336920722 | 0.58035336 |
| 2014 | Xiangtan | 0.998289057 | 0.298326571 | 0.545725344 |
| 2014 | Xiaogan | 0.999132587 | 0.337141309 | 0.580386826 |
| 2014 | Xinyu | 0.99961726 | 0.34390084 | 0.586318357 |
| 2014 | Xuzhou | 0.999806436 | 0.351959805 | 0.593204584 |
| 2014 | Xuancheng | 0.985039846 | 0.386648332 | 0.617141809 |
| 2014 | Yancheng | 0.997639135 | 0.366495598 | 0.604673756 |
| 2014 | Yangzhou | 0.994113746 | 0.305896548 | 0.551448967 |
| 2014 | Yibin | 0.982044517 | 0.31900423 | 0.559710956 |
| 2014 | Yichang | 0.994710275 | 0.300631781 | 0.54684689 |
| 2014 | Yichun | 0.993980524 | 0.295888794 | 0.542316973 |
| 2014 | Yiyang | 0.998349758 | 0.345696318 | 0.587474114 |
| 2014 | Yingtan | 0.987813024 | 0.291510677 | 0.536617223 |
| 2014 | yongzhou | 0.996061754 | 0.314887529 | 0.560042341 |
| 2014 | Yuxi | 0.998781532 | 0.292992445 | 0.540957894 |
| 2014 | yueyang | 0.992981543 | 0.309734024 | 0.554581075 |
| 2014 | Zhangjiajie | 0.995761311 | 0.391974755 | 0.624750587 |
| 2014 | Changsha | 0.995552585 | 0.302314075 | 0.548606926 |
| 2014 | Zhaotong | 0.98492385 | 0.246622662 | 0.492853469 |
| 2014 | Zhenjiang | 0.998480477 | 0.388366922 | 0.622717263 |
| 2014 | Chongqing | 0.99501306 | 0.394668264 | 0.626657862 |
| 2014 | Zhoushan | 0.979493022 | 0.360802583 | 0.594477596 |
| 2014 | Zhuzhou | 0.999852422 | 0.332392655 | 0.576492499 |
| 2014 | Ziyang | 0.976137947 | 0.331143198 | 0.568543263 |
| 2014 | Zigong | 0.998562445 | 0.315269312 | 0.561084748 |
| 2014 | Zunyi | 0.996167249 | 0.238667198 | 0.487598653 |
| 2015 | Anqing | 0.990990885 | 0.358924578 | 0.596398344 |
| 2015 | Anshun | 0.999358401 | 0.368341319 | 0.606716566 |
| 2015 | bazhong | 0.998866826 | 0.339447866 | 0.582291347 |
| 2015 | Bengbu | 0.991283842 | 0.383374501 | 0.616468125 |
| 2015 | Baoshan | 0.999509489 | 0.327630891 | 0.572250106 |
| 2015 | Bozhou | 0.999930806 | 0.346792911 | 0.588870881 |
| 2015 | Changde | 0.980117886 | 0.464867679 | 0.675000094 |
| 2015 | Changzhou | 0.996231683 | 0.346156796 | 0.587241319 |
| 2015 | chenzhou | 0.981156877 | 0.462472198 | 0.673615452 |
| 2015 | Chengdu | 0.988073014 | 0.369643032 | 0.60434618 |
| 2015 | Chizhou | 0.998671768 | 0.400899551 | 0.632745655 |
| 2015 | Chuzhou | 0.999997463 | 0.344676908 | 0.587091163 |
| 2015 | Dazhou | 0.990117351 | 0.370416598 | 0.605603749 |
| 2015 | Deyang | 0.988282945 | 0.396444211 | 0.625938537 |
| 2015 | Ezhou | 0.997656677 | 0.356668702 | 0.596517319 |
| 2015 | Fuzhou | 0.998487635 | 0.344198682 | 0.586240674 |
| 2015 | Fuyang | 0.992749415 | 0.369993747 | 0.60606194 |
| 2015 | Ganzhou | 0.999684164 | 0.354332406 | 0.595164259 |
| 2015 | Guangyuan | 0.988946192 | 0.432332875 | 0.653876097 |
| 2015 | Guiyang | 0.952202082 | 0.498918957 | 0.68925443 |
| 2015 | Hangzhou | 0.969658357 | 0.431602153 | 0.646920888 |
| 2015 | Hefei | 0.996372095 | 0.362011466 | 0.600581487 |
| 2015 | Hengyang | 0.974790977 | 0.439165303 | 0.654289213 |
| 2015 | Huzhou | 0.997877329 | 0.336580588 | 0.579539592 |
| 2015 | Huaihua | 0.995830737 | 0.399517526 | 0.63075497 |
| 2015 | Huai'an | 0.993932596 | 0.370482533 | 0.606823422 |
| 2015 | Huaibei | 0.988223574 | 0.383984865 | 0.616005597 |
| 2015 | Huainan | 0.999656811 | 0.385194465 | 0.62053386 |
| 2015 | Huanggang | 0.972780016 | 0.362417231 | 0.593761096 |
| 2015 | huangshan | 0.992497272 | 0.389357635 | 0.621640081 |
| 2015 | Huangshi | 0.993381148 | 0.332653814 | 0.574849569 |
| 2015 | Ji'an | 0.970963764 | 0.447413776 | 0.6591074 |
| 2015 | Jiaxing | 0.948923231 | 0.432860521 | 0.640898903 |
| 2015 | Jinhua | 0.993662704 | 0.370267851 | 0.60656521 |
| 2015 | Jingmen | 0.98588375 | 0.361558483 | 0.597038217 |
| 2015 | Jingzhou | 0.995818572 | 0.406775013 | 0.636454328 |
| 2015 | Jingdezhen | 0.989985316 | 0.374975647 | 0.609278577 |
| 2015 | Jiujiang | 0.97533074 | 0.434370288 | 0.650887621 |
| 2015 | Kunming | 0.988429596 | 0.361419502 | 0.597693677 |
| 2015 | Leshan | 0.99278636 | 0.380310123 | 0.614464566 |
| 2015 | Lijiang | 0.9959646 | 0.414267182 | 0.642335931 |
| 2015 | Lianyungang | 0.999550506 | 0.371662618 | 0.609504354 |
| 2015 | Liuan | 0.873970736 | 0.25867417 | 0.475472034 |
| 2015 | Liupanshui | 0.999828232 | 0.378613038 | 0.61526255 |
| 2015 | Loudi | 0.998393228 | 0.352652686 | 0.593368396 |
| 2015 | Luzhou | 0.997103088 | 0.365196493 | 0.603438937 |
| 2015 | Meishan | 0.995289741 | 0.393885917 | 0.62612348 |
| 2015 | Mianyang | 0.981340446 | 0.437699596 | 0.655387151 |
| 2015 | Nanchang | 0.956485408 | 0.462787573 | 0.665319142 |
| 2015 | Nanchong | 0.982503246 | 0.419111261 | 0.641699442 |
| 2015 | Nanjing | 0.999550664 | 0.35397512 | 0.5948244 |
| 2015 | Nantong | 0.969601384 | 0.468134036 | 0.673723541 |
| 2015 | Neijiang | 0.997246501 | 0.342785826 | 0.584672529 |
| 2015 | Ningbo | 0.986686554 | 0.365701004 | 0.600693152 |
| 2015 | Panzhihua | 0.995187141 | 0.379407302 | 0.614476418 |
| 2015 | Pingxiang | 0.998915423 | 0.343617009 | 0.585870574 |
| 2015 | Qujing | 0.887689116 | 0.35036664 | 0.557688671 |
| 2015 | Quzhou | 0.952713131 | 0.473887593 | 0.67192182 |
| 2015 | Shanghai | 0.998586631 | 0.368936039 | 0.60697166 |
| 2015 | Shangrao | 0.915448484 | 0.242607385 | 0.471269098 |
| 2015 | Shaoyang | 0.995342344 | 0.607376394 | 0.77752649 |
| 2015 | Shaoxing | 0.972217283 | 0.342000585 | 0.576627158 |
| 2015 | Shiyan | 0.962241536 | 0.444387156 | 0.653917257 |
| 2015 | Suzhou | 0.977643806 | 0.343669026 | 0.579642902 |
| 2015 | Suqian | 0.999074686 | 0.340466473 | 0.583225029 |
| 2015 | Suzhou | 0.99142393 | 0.36073798 | 0.598033666 |
| 2015 | Suizhou | 0.965467794 | 0.431818157 | 0.64568299 |
| 2015 | Taizhou | 0.976396239 | 0.394161999 | 0.620369481 |
| 2015 | Taizhou | 0.980740909 | 0.416060731 | 0.638786177 |
| 2015 | Tongling | 0.979058389 | 0.477947172 | 0.684060077 |
| 2015 | Wenzhou | 0.967873995 | 0.443910606 | 0.655476568 |
| 2015 | wuxi | 0.981509938 | 0.402842193 | 0.628803321 |
| 2015 | Wuhu | 0.961303718 | 0.452148488 | 0.659281444 |
| 2015 | Wuhan | 0.993189239 | 0.379047958 | 0.61356854 |
| 2015 | Xianning | 0.992594752 | 0.391900172 | 0.623697085 |
| 2015 | Xiangtan | 0.99751409 | 0.371531153 | 0.608775459 |
| 2015 | Xiaogan | 0.994822388 | 0.37304997 | 0.60919493 |
| 2015 | Xinyu | 0.989494274 | 0.40213049 | 0.630797763 |
| 2015 | Xuzhou | 0.989939505 | 0.382361567 | 0.615235582 |
| 2015 | Xuancheng | 0.993566414 | 0.399984661 | 0.630405683 |
| 2015 | Yancheng | 0.946645645 | 0.387880132 | 0.605957951 |
| 2015 | Yangzhou | 0.997863176 | 0.358893297 | 0.598436634 |
| 2015 | Yibin | 0.983553649 | 0.403523947 | 0.62999004 |
| 2015 | Yichang | 0.995188997 | 0.347032959 | 0.587676256 |
| 2015 | Yichun | 0.99784413 | 0.347228498 | 0.588625448 |
| 2015 | Yiyang | 0.998506943 | 0.392963715 | 0.62640003 |
| 2015 | Yingtan | 0.999735627 | 0.345810214 | 0.587978564 |
| 2015 | Yongzhou | 0.999102383 | 0.395370031 | 0.6285023 |
| 2015 | Yuxi | 0.980934162 | 0.488579642 | 0.692289291 |
| 2015 | Yueyang | 0.997907244 | 0.355973707 | 0.596010689 |
| 2015 | Zhangjiajie | 0.980591616 | 0.453866942 | 0.667126763 |
| 2015 | Changsha | 0.99755253 | 0.350299647 | 0.591136447 |
| 2015 | Zhaotong | 0.984189237 | 0.367133797 | 0.601106589 |
| 2015 | Zhenjiang | 0.976430264 | 0.430201974 | 0.648122077 |
| 2015 | Chongqing | 0.98589355 | 0.398070559 | 0.626462446 |
| 2015 | Zhoushan | 0.964075971 | 0.424050526 | 0.639387928 |
| 2015 | Zhuzhou | 0.987156113 | 0.380855995 | 0.613159297 |
| 2015 | Ziyang | 0.999925152 | 0.358998946 | 0.599142784 |
| 2015 | Zigong | 0.99155353 | 0.358427676 | 0.596154533 |
| 2015 | Zunyi | 0.970344313 | 0.49508454 | 0.693110718 |
| 2016 | Anqing | 0.983171702 | 0.426822127 | 0.64779583 |
| 2016 | Anshun | 0.98960657 | 0.412773827 | 0.639127289 |
| 2016 | Bazhong | 0.983519431 | 0.355572227 | 0.591364688 |
| 2016 | Bengbu | 0.982939809 | 0.429952655 | 0.65009044 |
| 2016 | Baoshan | 0.99718591 | 0.404303472 | 0.634953325 |
| 2016 | Bozhou | 0.993128359 | 0.407148877 | 0.635886071 |
| 2016 | Changde | 0.966066133 | 0.520837917 | 0.709340449 |
| 2016 | Changzhou | 0.990965149 | 0.418539225 | 0.644016914 |
| 2016 | Chenzhou | 0.994648946 | 0.569142388 | 0.752394096 |
| 2016 | Chengdu | 0.973852783 | 0.428515553 | 0.64599618 |
| 2016 | Chizhou | 0.981057966 | 0.432719961 | 0.651554576 |
| 2016 | Chuzhou | 0.994802104 | 0.397734132 | 0.62902047 |
| 2016 | Dazhou | 0.978710604 | 0.43129666 | 0.649703482 |
| 2016 | Deyang | 0.978683086 | 0.475668924 | 0.682296952 |
| 2016 | Ezhou | 0.98195832 | 0.401747192 | 0.628091552 |
| 2016 | Fuzhou | 0.986952589 | 0.40181135 | 0.62973705 |
| 2016 | Fuyang | 0.980409031 | 0.428324085 | 0.648022223 |
| 2016 | Ganzhou | 0.993928785 | 0.415964439 | 0.642992247 |
| 2016 | Guangyuan | 0.968401454 | 0.466648002 | 0.672237015 |
| 2016 | Guiyang | 0.935126847 | 0.525834931 | 0.701229179 |
| 2016 | Hangzhou | 0.955461936 | 0.477448649 | 0.675413955 |
| 2016 | Hefei | 0.985188502 | 0.393849167 | 0.622909039 |
| 2016 | Hengyang | 0.962132094 | 0.47324947 | 0.674780337 |
| 2016 | Huzhou | 0.989569706 | 0.397531275 | 0.627204039 |
| 2016 | Huaihua | 0.976612394 | 0.459914634 | 0.670192757 |
| 2016 | Huai'an | 0.963923563 | 0.494048329 | 0.690090447 |
| 2016 | Huaibei | 0.98248911 | 0.408077731 | 0.633191856 |
| 2016 | Huainan | 0.96889493 | 0.39603982 | 0.619452156 |
| 2016 | Huanggang | 0.992002968 | 0.464914152 | 0.67911429 |
| 2016 | huangshan | 0.975981736 | 0.436763332 | 0.652895884 |
| 2016 | Huangshi | 0.989543142 | 0.412145318 | 0.638620054 |
| 2016 | Ji'an | 0.966738559 | 0.486519461 | 0.685811288 |
| 2016 | Jiaxing | 0.967578889 | 0.492220548 | 0.690117534 |
| 2016 | Jinhua | 0.886374652 | 0.348656001 | 0.55591352 |
| 2016 | Jingmen | 0.980827502 | 0.41795311 | 0.640265496 |
| 2016 | Jingzhou | 0.954936738 | 0.400012249 | 0.618050477 |
| 2016 | Jingdezhen | 0.982844711 | 0.438191439 | 0.656257676 |
| 2016 | Jiujiang | 0.968088884 | 0.492253513 | 0.6903225 |
| 2016 | Kunming | 0.974324832 | 0.406086411 | 0.629015162 |
| 2016 | Leshan | 0.982925867 | 0.431291997 | 0.651097581 |
| 2016 | Lijiang | 0.973309848 | 0.474358172 | 0.679483245 |
| 2016 | Lianyungang | 0.990069559 | 0.434608549 | 0.655966992 |
| 2016 | Liuan | 0.996938413 | 0.418492984 | 0.645919291 |
| 2016 | Liupanshui | 0.999912466 | 0.446729809 | 0.668349239 |
| 2016 | Loudi | 0.986717078 | 0.400221126 | 0.628414688 |
| 2016 | Luzhou | 0.98525197 | 0.406898214 | 0.633164486 |
| 2016 | Meishan | 0.979016796 | 0.412558555 | 0.635532655 |
| 2016 | Mianyang | 0.951241587 | 0.461308547 | 0.662431789 |
| 2016 | Nanchang | 0.934855423 | 0.518336817 | 0.696110612 |
| 2016 | Nanchong | 0.956175331 | 0.461739101 | 0.664457326 |
| 2016 | Nanjing | 0.989045144 | 0.3919352 | 0.62260871 |
| 2016 | Nantong | 0.958813707 | 0.498847292 | 0.691593538 |
| 2016 | Neijiang | 0.988831652 | 0.403719138 | 0.631830881 |
| 2016 | Ningbo | 0.981040535 | 0.424105197 | 0.645030534 |
| 2016 | Panzhihua | 0.980880336 | 0.431846732 | 0.650837897 |
| 2016 | Pingxiang | 0.991054881 | 0.404817246 | 0.633400433 |
| 2016 | Qujing | 0.983735192 | 0.484322228 | 0.690249824 |
| 2016 | Quzhou | 0.945670693 | 0.51427845 | 0.697379422 |
| 2016 | Shanghai | 0.992133102 | 0.425593209 | 0.649803902 |
| 2016 | Shangrao | 0.995119209 | 0.394946734 | 0.62691234 |
| 2016 | Shaoyang | 0.825524481 | 0.390421536 | 0.567716951 |
| 2016 | Shaoxing | 0.974827011 | 0.433331969 | 0.649941311 |
| 2016 | Shiyan | 0.944760414 | 0.507542458 | 0.692463733 |
| 2016 | Suzhou | 0.997775478 | 0.51997595 | 0.720291088 |
| 2016 | Suqian | 0.99095074 | 0.406783451 | 0.634903427 |
| 2016 | Suzhou | 0.980152878 | 0.428609228 | 0.648153199 |
| 2016 | Suizhou | 0.960239092 | 0.471682705 | 0.672999385 |
| 2016 | Taizhou | 0.953266354 | 0.467746606 | 0.667747783 |
| 2016 | Taizhou | 0.986206751 | 0.440015636 | 0.658746075 |
| 2016 | Tongling | 0.927401588 | 0.469525823 | 0.659878014 |
| 2016 | Wenzhou | 0.942131785 | 0.485976369 | 0.676648937 |
| 2016 | Wuxi | 0.979269649 | 0.463745858 | 0.673893347 |
| 2016 | Wuhu | 0.950031206 | 0.500398439 | 0.689488312 |
| 2016 | Wuhan | 0.970979161 | 0.433230535 | 0.648581392 |
| 2016 | Xianning | 0.978624508 | 0.430447073 | 0.64903471 |
| 2016 | Xiangtan | 0.978471174 | 0.413903124 | 0.636390035 |
| 2016 | Xiaogan | 0.966682956 | 0.401178778 | 0.622746086 |
| 2016 | Xinyu | 0.973445468 | 0.465428017 | 0.673103851 |
| 2016 | Xuzhou | 0.983634522 | 0.42717454 | 0.648215723 |
| 2016 | Xuancheng | 0.976221087 | 0.474599268 | 0.68067159 |
| 2016 | Yancheng | 0.95795101 | 0.493085361 | 0.687278415 |
| 2016 | Yangzhou | 0.989824983 | 0.401922158 | 0.630739719 |
| 2016 | Yibin | 0.97737179 | 0.459772729 | 0.67034983 |
| 2016 | Yichang | 0.990360023 | 0.435090841 | 0.656427129 |
| 2016 | Yichun | 0.995952714 | 0.389371127 | 0.62273207 |
| 2016 | Yiyang | 0.985420865 | 0.441637436 | 0.659695948 |
| 2016 | Yingtan | 0.989518523 | 0.398486377 | 0.627940802 |
| 2016 | Yongzhou | 0.987702614 | 0.443306606 | 0.661706199 |
| 2016 | Yuxi | 0.807814441 | 0.299611975 | 0.491966341 |
| 2016 | Yueyang | 0.99108521 | 0.420363561 | 0.645458061 |
| 2016 | Zhangjiajie | 0.962776712 | 0.489990267 | 0.686841479 |
| 2016 | Changsha | 0.991711802 | 0.406259731 | 0.63473819 |
| 2016 | Zhaotong | 0.947453014 | 0.306486413 | 0.538870555 |
| 2016 | Zhenjiang | 0.97545058 | 0.507574775 | 0.703643453 |
| 2016 | Chongqing | 0.988636009 | 0.479244129 | 0.688329865 |
| 2016 | Zhoushan | 0.965943296 | 0.490230454 | 0.688138664 |
| 2016 | Zhuzhou | 0.980313235 | 0.42069081 | 0.642190602 |
| 2016 | Ziyang | 0.966873744 | 0.375019054 | 0.602159511 |
| 2016 | Zigong | 0.994140494 | 0.42989825 | 0.653742502 |
| 2016 | Zunyi | 0.842062614 | 0.315935616 | 0.5157883 |
| 2017 | Anqing | 0.96628758 | 0.475906713 | 0.678131806 |
| 2017 | Anshun | 0.960091602 | 0.448739846 | 0.656377451 |
| 2017 | Bazhong | 0.956496542 | 0.397465842 | 0.616583087 |
| 2017 | Bengbu | 0.955720841 | 0.475198875 | 0.673912063 |
| 2017 | Baoshan | 0.914936517 | 0.382559229 | 0.591622691 |
| 2017 | Bozhou | 0.945398261 | 0.420281822 | 0.630344115 |
| 2017 | Changde | 0.863011203 | 0.507471291 | 0.661780484 |
| 2017 | Changzhou | 0.994563995 | 0.526056074 | 0.723323185 |
| 2017 | chenzhou | 0.769348342 | 0.450927388 | 0.588999353 |
| 2017 | Chengdu | 0.944247288 | 0.463250548 | 0.661379674 |
| 2017 | Chizhou | 0.935738417 | 0.456299194 | 0.65343453 |
| 2017 | Chuzhou | 0.935743862 | 0.388074877 | 0.602609893 |
| 2017 | Dazhou | 0.908936679 | 0.434497422 | 0.628435075 |
| 2017 | Deyang | 0.895396014 | 0.461319029 | 0.642699945 |
| 2017 | Ezhou | 0.969309813 | 0.465227519 | 0.67152781 |
| 2017 | Fuzhou | 0.92489048 | 0.41755687 | 0.621445391 |
| 2017 | Fuyang | 0.941553805 | 0.467696192 | 0.663597113 |
| 2017 | Ganzhou | 0.951730996 | 0.418045785 | 0.630767097 |
| 2017 | Guangyuan | 0.931634088 | 0.515776602 | 0.693191939 |
| 2017 | Guiyang | 0.902260623 | 0.601235593 | 0.736526443 |
| 2017 | Hangzhou | 0.91923193 | 0.536726918 | 0.70240766 |
| 2017 | Hefei | 0.951831485 | 0.439046914 | 0.64645083 |
| 2017 | Hengyang | 0.918841519 | 0.518147527 | 0.68999671 |
| 2017 | Huzhou | 0.942984565 | 0.414921002 | 0.625511072 |
| 2017 | Huaihua | 0.890568235 | 0.467998496 | 0.645588564 |
| 2017 | Huai'an | 0.962065645 | 0.480607007 | 0.679981978 |
| 2017 | Huaibei | 0.948264054 | 0.467354353 | 0.665714153 |
| 2017 | Huainan | 0.95632655 | 0.458160056 | 0.661929472 |
| 2017 | Huanggang | 0.75143568 | 0.383404753 | 0.536753213 |
| 2017 | huangshan | 0.940169803 | 0.484024704 | 0.674585362 |
| 2017 | Huangshi | 0.960700344 | 0.441939305 | 0.651591316 |
| 2017 | Ji'an | 0.845279862 | 0.479205704 | 0.636445545 |
| 2017 | Jiaxing | 0.856983928 | 0.487444998 | 0.64632231 |
| 2017 | Jinhua | 0.83752937 | 0.392368926 | 0.573254306 |
| 2017 | Jingmen | 0.941200121 | 0.4615064 | 0.659067432 |
| 2017 | Jingzhou | 0.000251542 | 0.316089524 | 0.00891682 |
| 2017 | Jingdezhen | 0.918048749 | 0.449830031 | 0.642624227 |
| 2017 | Jiujiang | 0.911010133 | 0.504242243 | 0.677768244 |
| 2017 | Kunming | 0.920665733 | 0.426597359 | 0.626700542 |
| 2017 | Leshan | 0.941406997 | 0.455973415 | 0.655176742 |
| 2017 | Lijiang | 0.870515488 | 0.450029354 | 0.625905363 |
| 2017 | Lianyungang | 0.779498523 | 0.36842413 | 0.535897439 |
| 2017 | Liuan | 0.926120189 | 0.406049846 | 0.613229941 |
| 2017 | Liupanshui | 0.912404972 | 0.3972499 | 0.602040517 |
| 2017 | Loudi | 0.957712721 | 0.43442159 | 0.645020219 |
| 2017 | Luzhou | 0.977029503 | 0.473718377 | 0.680321123 |
| 2017 | Meishan | 0.922590174 | 0.457857313 | 0.649934349 |
| 2017 | Mianyang | 0.924642955 | 0.533058303 | 0.702060257 |
| 2017 | Nanchang | 0.89775758 | 0.561174647 | 0.709787851 |
| 2017 | Nanchong | 0.925757998 | 0.514684123 | 0.690270196 |
| 2017 | Nanjing | 0.971070799 | 0.443155445 | 0.655999476 |
| 2017 | Nantong | 0.875783793 | 0.510515464 | 0.668656242 |
| 2017 | Neijiang | 0.924068692 | 0.406142232 | 0.612620047 |
| 2017 | Ningbo | 0.954911858 | 0.461885095 | 0.664123147 |
| 2017 | Panzhihua | 0.96549658 | 0.478062648 | 0.679387851 |
| 2017 | Pingxiang | 0.962417731 | 0.436183749 | 0.647912783 |
| 2017 | Qujing | 0.817541888 | 0.439000248 | 0.599083543 |
| 2017 | Quzhou | 0.893911225 | 0.567878011 | 0.712483353 |
| 2017 | Shanghai | 0.973060182 | 0.475077878 | 0.679911293 |
| 2017 | Shangrao | 0.927336169 | 0.387175052 | 0.599200658 |
| 2017 | Shaoyang | 0.85937534 | 0.468217538 | 0.634330045 |
| 2017 | Shaoxing | 0.947707247 | 0.472782114 | 0.669372121 |
| 2017 | Shiyan | 0.897310495 | 0.548901646 | 0.701808526 |
| 2017 | Suzhou | 0.924747958 | 0.484720952 | 0.6695108 |
| 2017 | Suqian | 0.966326724 | 0.442231999 | 0.653712933 |
| 2017 | Suzhou | 0.935146929 | 0.44305773 | 0.643680104 |
| 2017 | Suizhou | 0.90997339 | 0.503292464 | 0.676744228 |
| 2017 | Taizhou | 0.900455768 | 0.496716014 | 0.668783074 |
| 2017 | Taizhou | 0.989362112 | 0.528185758 | 0.722887942 |
| 2017 | Tongling | 0.935855959 | 0.564467283 | 0.726815018 |
| 2017 | Wenzhou | 0.86545484 | 0.508479943 | 0.663375028 |
| 2017 | Wuxi | 0.921096506 | 0.497941297 | 0.677238502 |
| 2017 | Wuhu | 0.906947392 | 0.552512347 | 0.707883912 |
| 2017 | Wuhan | 0.950770458 | 0.472737182 | 0.670421171 |
| 2017 | Xianning | 0.921180941 | 0.453618786 | 0.646424767 |
| 2017 | Xiangtan | 0.973643127 | 0.488361751 | 0.689557875 |
| 2017 | Xiaogan | 0.869825355 | 0.414634199 | 0.600549198 |
| 2017 | Xinyu | 0.943860139 | 0.513332113 | 0.696070197 |
| 2017 | Xuzhou | 0.954525395 | 0.478861845 | 0.676081202 |
| 2017 | Xuancheng | 0.882035384 | 0.46476633 | 0.640265842 |
| 2017 | Yancheng | 0.922088645 | 0.526448006 | 0.696729308 |
| 2017 | Yangzhou | 0.867480846 | 0.380596338 | 0.574595539 |
| 2017 | Yibin | 0.931786769 | 0.477970879 | 0.667358181 |
| 2017 | Yichang | 0.934902104 | 0.426016343 | 0.631097121 |
| 2017 | Yichun | 0.906334874 | 0.386368291 | 0.591759289 |
| 2017 | Yiyang | 0.926554241 | 0.439645388 | 0.638243918 |
| 2017 | Yingtan | 0.913814668 | 0.394964155 | 0.600769538 |
| 2017 | yongzhou | 0.901253879 | 0.416961412 | 0.613015571 |
| 2017 | Yuxi | 0.797504649 | 0.36171784 | 0.537095577 |
| 2017 | Yueyang | 0.946325044 | 0.441051162 | 0.646047801 |
| 2017 | Zhangjiajie | 0.919701566 | 0.531728921 | 0.699308173 |
| 2017 | Changsha | 0.945386255 | 0.429944536 | 0.637545022 |
| 2017 | Zhaotong | 0.9203709 | 0.353834196 | 0.570665136 |
| 2017 | Zhenjiang | 0.90838926 | 0.517465842 | 0.685609519 |
| 2017 | Chongqing | 0.948141971 | 0.500809211 | 0.689085069 |
| 2017 | Zhoushan | 0.965784907 | 0.568669821 | 0.741088881 |
| 2017 | Zhuzhou | 0.94668602 | 0.462205812 | 0.66148604 |
| 2017 | Ziyang | 0.951290504 | 0.441517606 | 0.648082947 |
| 2017 | Zigong | 0.951865242 | 0.443032783 | 0.649390104 |
| 2017 | Zunyi | 0.676253263 | 0.330770292 | 0.472952946 |
| 2018 | Anqing | 0.937413123 | 0.483441678 | 0.673189849 |
| 2018 | Anshun | 0.972419475 | 0.493189148 | 0.692522008 |
| 2018 | Bazhong | 0.966835776 | 0.427443918 | 0.642859295 |
| 2018 | Bengbu | 0.940600785 | 0.503951983 | 0.688489383 |
| 2018 | Baoshan | 0.923046041 | 0.416797365 | 0.620260556 |
| 2018 | Bozhou | 0.952680307 | 0.463511219 | 0.664513364 |
| 2018 | Changde | 0.941826886 | 0.62701718 | 0.76846707 |
| 2018 | Changzhou | 0.952140231 | 0.473326285 | 0.671321829 |
| 2018 | Chenzhou | 0.933101276 | 0.580022097 | 0.735676124 |
| 2018 | Chengdu | 0.942316014 | 0.498459137 | 0.685351025 |
| 2018 | Chizhou | 0.941014054 | 0.501879991 | 0.68722349 |
| 2018 | Chuzhou | 0.950270002 | 0.424250012 | 0.634942565 |
| 2018 | Dazhou | 0.943191621 | 0.475676612 | 0.669816538 |
| 2018 | Deyang | 0.938176557 | 0.539722561 | 0.711586294 |
| 2018 | Ezhou | 0.953182913 | 0.478535985 | 0.675375691 |
| 2018 | Fuzhou | 0.962341604 | 0.48948588 | 0.686332738 |
| 2018 | Fuyang | 0.934587922 | 0.496957562 | 0.681506079 |
| 2018 | Ganzhou | 0.972679028 | 0.465487763 | 0.672881999 |
| 2018 | Guangyuan | 0.934068945 | 0.5467269 | 0.714619213 |
| 2018 | Guiyang | 0.885215994 | 0.633426808 | 0.748812087 |
| 2018 | Hangzhou | 0.906970292 | 0.570474018 | 0.719307297 |
| 2018 | Hefei | 0.953868838 | 0.471306472 | 0.670495755 |
| 2018 | Hengyang | 0.92105762 | 0.576226317 | 0.728517426 |
| 2018 | Huzhou | 0.957260264 | 0.454740394 | 0.659776409 |
| 2018 | Huaihua | 0.933110251 | 0.546090823 | 0.713836778 |
| 2018 | Huai'an | 0.971353609 | 0.515711956 | 0.707770209 |
| 2018 | Huaibei | 0.940619682 | 0.489420488 | 0.678497269 |
| 2018 | Huainan | 0.951556796 | 0.489433745 | 0.682439745 |
| 2018 | Huanggang | 0.933200262 | 0.506003633 | 0.687170083 |
| 2018 | huangshan | 0.934372402 | 0.513790486 | 0.69287203 |
| 2018 | Huangshi | 0.95089612 | 0.467787652 | 0.666946372 |
| 2018 | Ji'an | 0.921561071 | 0.584629359 | 0.734010666 |
| 2018 | Jiaxing | 0.903936945 | 0.580488193 | 0.724378854 |
| 2018 | Jinhua | 0.973895506 | 0.530889879 | 0.719048863 |
| 2018 | Jingmen | 0.944562547 | 0.510804539 | 0.694612724 |
| 2018 | Jingzhou | 0.981910823 | 0.840799661 | 0.90861999 |
| 2018 | Jingdezhen | 0.950511334 | 0.51594595 | 0.700294562 |
| 2018 | Jiujiang | 0.938471987 | 0.566499554 | 0.729139193 |
| 2018 | Kunming | 0.979599476 | 0.524406236 | 0.716734312 |
| 2018 | Leshan | 0.957507241 | 0.50008383 | 0.691978243 |
| 2018 | Lijiang | 0.904023034 | 0.509108397 | 0.67841412 |
| 2018 | Lianyungang | 0.995406081 | 0.598173724 | 0.771638362 |
| 2018 | Liuan | 0.959910229 | 0.461970325 | 0.665920446 |
| 2018 | Liupanshui | 0.971218827 | 0.477622634 | 0.681084498 |
| 2018 | Loudi | 0.966285598 | 0.471431578 | 0.674935215 |
| 2018 | Luzhou | 0.930932779 | 0.449385732 | 0.646798198 |
| 2018 | Meishan | 0.947621696 | 0.497594186 | 0.686681183 |
| 2018 | Mianyang | 0.909873959 | 0.55289931 | 0.70927335 |
| 2018 | Nanchang | 0.885603118 | 0.597447558 | 0.72739358 |
| 2018 | Nanchong | 0.925742725 | 0.565930346 | 0.723813443 |
| 2018 | Nanjing | 0.958503623 | 0.456747596 | 0.661660204 |
| 2018 | Nantong | 0.907316616 | 0.575871978 | 0.72284038 |
| 2018 | Neijiang | 0.948111846 | 0.447757328 | 0.651555083 |
| 2018 | Ningbo | 0.965537792 | 0.500768928 | 0.695349786 |
| 2018 | Panzhihua | 0.946628364 | 0.480644125 | 0.674530475 |
| 2018 | Pingxiang | 0.962933515 | 0.477040037 | 0.677759426 |
| 2018 | Qujing | 0.881066747 | 0.519529844 | 0.6765652 |
| 2018 | Quzhou | 0.900627135 | 0.615947075 | 0.744807794 |
| 2018 | Shanghai | 0.953026564 | 0.487608853 | 0.681692152 |
| 2018 | Shangrao | 0.978309318 | 0.461057772 | 0.671607858 |
| 2018 | Shaoyang | 0.927480285 | 0.560937225 | 0.721289274 |
| 2018 | Shaoxing | 0.931850428 | 0.492555873 | 0.677486828 |
| 2018 | Shiyan | 0.891800262 | 0.583267864 | 0.721220101 |
| 2018 | Suzhou | 0.941361354 | 0.540750824 | 0.713471743 |
| 2018 | Suqian | 0.962307045 | 0.467967946 | 0.67106546 |
| 2018 | Suzhou | 0.977131075 | 0.53730324 | 0.724579666 |
| 2018 | Suizhou | 0.953305213 | 0.597134248 | 0.75448737 |
| 2018 | Taizhou | 0.918394247 | 0.547292671 | 0.708964344 |
| 2018 | Taizhou | 0.929988263 | 0.493296758 | 0.677318385 |
| 2018 | Tongling | 0.9079618 | 0.575298065 | 0.722736928 |
| 2018 | Wenzhou | 0.904740387 | 0.578182764 | 0.723260187 |
| 2018 | wuxi | 0.949135579 | 0.564987315 | 0.732290627 |
| 2018 | Wuhu | 0.890767536 | 0.581579868 | 0.719758617 |
| 2018 | Wuhan | 0.949326388 | 0.514854304 | 0.699117141 |
| 2018 | Xianning | 0.950847617 | 0.50708731 | 0.694379407 |
| 2018 | Xiangtan | 0.942146525 | 0.488703259 | 0.678549982 |
| 2018 | Xiaogan | 0.943251092 | 0.501877578 | 0.688038207 |
| 2018 | Xinyu | 0.929639696 | 0.539563799 | 0.708237196 |
| 2018 | Xuzhou | 0.963970901 | 0.536833094 | 0.719368808 |
| 2018 | Xuancheng | 0.911198593 | 0.51554878 | 0.685395742 |
| 2018 | Yancheng | 0.934961699 | 0.573717326 | 0.73239588 |
| 2018 | Yangzhou | 0.992875177 | 0.539056124 | 0.731584202 |
| 2018 | Yibin | 0.922862275 | 0.506043441 | 0.683380129 |
| 2018 | Yichang | 0.948644682 | 0.479522261 | 0.674459963 |
| 2018 | Yichun | 0.968133863 | 0.471775153 | 0.675826532 |
| 2018 | Yiyang | 0.974977217 | 0.53623671 | 0.723061944 |
| 2018 | Yingtan | 0.959841686 | 0.460171097 | 0.664598677 |
| 2018 | yongzhou | 0.960637924 | 0.498205526 | 0.691805697 |
| 2018 | Yuxi | 0.998225229 | 0.584505359 | 0.763850768 |
| 2018 | Yueyang | 0.976450135 | 0.507542167 | 0.703981262 |
| 2018 | Zhangjiajie | 0.923493176 | 0.568568644 | 0.724616632 |
| 2018 | Changsha | 0.970130216 | 0.488332732 | 0.688292335 |
| 2018 | Zhaotong | 0.959706766 | 0.417156177 | 0.632730279 |
| 2018 | Zhenjiang | 0.936599842 | 0.58734507 | 0.741692187 |
| 2018 | Chongqing | 0.936838375 | 0.512768029 | 0.693095064 |
| 2018 | Zhoushan | 0.97939708 | 0.648801951 | 0.797141604 |
| 2018 | Zhuzhou | 0.949675454 | 0.501893223 | 0.690388061 |
| 2018 | Ziyang | 0.94329173 | 0.459756932 | 0.658547578 |
| 2018 | Zigong | 0.981633773 | 0.514237821 | 0.71048801 |
| 2018 | Zunyi | 0.989389254 | 0.536847315 | 0.728801046 |
| 2019 | Anqing | 0.957441849 | 0.54286441 | 0.720944592 |
| 2019 | Anshun | 0.942428045 | 0.48460577 | 0.675800317 |
| 2019 | Bazhong | 0.975662075 | 0.471698172 | 0.678393704 |
| 2019 | Bengbu | 0.946827779 | 0.549517906 | 0.721317419 |
| 2019 | Baoshan | 0.994831479 | 0.55428747 | 0.742578362 |
| 2019 | Bozhou | 0.978553182 | 0.541604585 | 0.728003359 |
| 2019 | Changde | 0.863938607 | 0.592879926 | 0.715689777 |
| 2019 | Changzhou | 0.946347721 | 0.485556168 | 0.677867961 |
| 2019 | chenzhou | 0.879686027 | 0.571829371 | 0.709246296 |
| 2019 | Chengdu | 0.930826179 | 0.521908178 | 0.696997701 |
| 2019 | Chizhou | 0.947260259 | 0.547890071 | 0.720412722 |
| 2019 | Chuzhou | 0.994369136 | 0.542767043 | 0.734650117 |
| 2019 | Dazhou | 0.963667706 | 0.541810135 | 0.722582126 |
| 2019 | Deyang | 0.933786608 | 0.570514559 | 0.729889619 |
| 2019 | Ezhou | 0.947793087 | 0.508855262 | 0.694470662 |
| 2019 | Fuzhou | 0.940404849 | 0.503173137 | 0.687885498 |
| 2019 | Fuyang | 0.968429422 | 0.582127398 | 0.750832405 |
| 2019 | Ganzhou | 0.966437546 | 0.493067447 | 0.690303479 |
| 2019 | Guangyuan | 0.929383789 | 0.578191445 | 0.733049627 |
| 2019 | Guiyang | 0.849522723 | 0.654642186 | 0.74574353 |
| 2019 | Hangzhou | 0.940629057 | 0.650042614 | 0.781952026 |
| 2019 | Hefei | 0.954090067 | 0.505263757 | 0.694310545 |
| 2019 | Hengyang | 0.896652789 | 0.592073195 | 0.728617925 |
| 2019 | Huzhou | 0.954465209 | 0.485525554 | 0.680747567 |
| 2019 | Huaihua | 0.902256851 | 0.553621377 | 0.70675928 |
| 2019 | Huai'an | 0.946187578 | 0.522779721 | 0.703311935 |
| 2019 | Huaibei | 0.941524518 | 0.52526166 | 0.703240166 |
| 2019 | Huainan | 0.952291122 | 0.529363851 | 0.710005983 |
| 2019 | Huanggang | 0.929287742 | 0.53986138 | 0.708298357 |
| 2019 | huangshan | 0.937056025 | 0.550647506 | 0.718322743 |
| 2019 | Huangshi | 0.946844557 | 0.503636705 | 0.690554613 |
| 2019 | Ji'an | 0.901197474 | 0.605438082 | 0.738660456 |
| 2019 | Jiaxing | 0.9022579 | 0.626855385 | 0.752054003 |
| 2019 | Jinhua | 0.953202975 | 0.533750294 | 0.713282811 |
| 2019 | Jingmen | 0.928676887 | 0.526220109 | 0.699062553 |
| 2019 | Jingzhou | 0.944365413 | 0.548732211 | 0.719863682 |
| 2019 | Jingdezhen | 0.952516683 | 0.552120893 | 0.725192638 |
| 2019 | Jiujiang | 0.91881328 | 0.581479946 | 0.730938778 |
| 2019 | Kunming | 0.958119142 | 0.520999142 | 0.706526186 |
| 2019 | Leshan | 0.95150196 | 0.514791694 | 0.699875207 |
| 2019 | Lijiang | 0.950102518 | 0.60612079 | 0.758865527 |
| 2019 | Lianyungang | 0.953641636 | 0.536924726 | 0.715565353 |
| 2019 | Liuan | 0.966916934 | 0.504857855 | 0.698681336 |
| 2019 | Liupanshui | 0.895184833 | 0.447461862 | 0.632898943 |
| 2019 | Loudi | 0.950053966 | 0.480872885 | 0.675910639 |
| 2019 | Luzhou | 0.952678601 | 0.503594856 | 0.692650015 |
| 2019 | Meishan | 0.918227408 | 0.498828094 | 0.676784772 |
| 2019 | Mianyang | 0.932576745 | 0.611934931 | 0.755431192 |
| 2019 | Nanchang | 0.871108983 | 0.623221061 | 0.736813046 |
| 2019 | Nanchong | 0.917873156 | 0.601804931 | 0.74322311 |
| 2019 | Nanjing | 0.973681744 | 0.506872765 | 0.702518866 |
| 2019 | Nantong | 0.975541452 | 0.716504485 | 0.836050133 |
| 2019 | Neijiang | 0.939085451 | 0.467271319 | 0.662425616 |
| 2019 | Ningbo | 0.961007151 | 0.522543464 | 0.708638134 |
| 2019 | Panzhihua | 0.90942941 | 0.476887484 | 0.658555619 |
| 2019 | Pingxiang | 0.930191498 | 0.465728636 | 0.658192082 |
| 2019 | Qujing | 0.963595933 | 0.648744635 | 0.79065017 |
| 2019 | Quzhou | 0.86988981 | 0.636121779 | 0.743878924 |
| 2019 | Shanghai | 0.967104052 | 0.542085257 | 0.724053071 |
| 2019 | Shangrao | 0.961230935 | 0.473948878 | 0.67496246 |
| 2019 | Shaoyang | 0.914770031 | 0.602876468 | 0.742625966 |
| 2019 | Shaoxing | 0.92827209 | 0.525017703 | 0.698111223 |
| 2019 | Shiyan | 0.893677913 | 0.627041765 | 0.748580908 |
| 2019 | Suzhou | 0.914556598 | 0.551847914 | 0.7104197 |
| 2019 | Suqian | 0.960122264 | 0.504798605 | 0.696181283 |
| 2019 | Suzhou | 0.960965533 | 0.541190769 | 0.721155791 |
| 2019 | Suizhou | 0.931645407 | 0.612752028 | 0.755557816 |
| 2019 | Taizhou | 0.899674796 | 0.570726779 | 0.716567162 |
| 2019 | Taizhou | 0.761888376 | 0.446206523 | 0.583060514 |
| 2019 | Tongling | 0.825237995 | 0.553129654 | 0.675620905 |
| 2019 | Wenzhou | 0.907679631 | 0.617106226 | 0.748421507 |
| 2019 | Wuxi | 0.905490326 | 0.570934235 | 0.719010032 |
| 2019 | Wuhu | 0.911160151 | 0.638000259 | 0.762443711 |
| 2019 | Wuhan | 0.95484275 | 0.553626701 | 0.727067013 |
| 2019 | Xianning | 0.944620238 | 0.529052576 | 0.706932649 |
| 2019 | Xiangtan | 0.932126784 | 0.519281665 | 0.695727208 |
| 2019 | Xiaogan | 0.95118291 | 0.529710341 | 0.709824925 |
| 2019 | Xinyu | 0.873108941 | 0.536671835 | 0.684523905 |
| 2019 | Xuzhou | 0.952950262 | 0.559199997 | 0.729993002 |
| 2019 | Xuancheng | 0.931081775 | 0.569205448 | 0.727995068 |
| 2019 | Yancheng | 0.96024581 | 0.647762384 | 0.788676813 |
| 2019 | Yangzhou | 0.969170994 | 0.516418027 | 0.707458389 |
| 2019 | Yibin | 0.94957028 | 0.571534432 | 0.736689969 |
| 2019 | Yichang | 0.93808956 | 0.496313642 | 0.682339099 |
| 2019 | Yichun | 0.969137339 | 0.505053268 | 0.699618454 |
| 2019 | Yiyang | 0.897437881 | 0.483221844 | 0.658529869 |
| 2019 | Yingtan | 0.966628135 | 0.50500152 | 0.698676375 |
| 2019 | Yongzhou | 0.948605093 | 0.509645797 | 0.695307557 |
| 2019 | Yuxi | 0.999979579 | 0.666018257 | 0.816091083 |
| 2019 | Yueyang | 0.908662731 | 0.464640182 | 0.649770126 |
| 2019 | Zhangjiajie | 0.844681228 | 0.551695436 | 0.682646892 |
| 2019 | Changsha | 0.902557564 | 0.453566243 | 0.639820009 |
| 2019 | Zhaotong | 0.980005654 | 0.471110569 | 0.679478492 |
| 2019 | Zhenjiang | 0.89276326 | 0.580527852 | 0.719912452 |
| 2019 | Chongqing | 0.943773138 | 0.55738343 | 0.725288569 |
| 2019 | Zhoushan | 0.763738395 | 0.506466633 | 0.621938914 |
| 2019 | Zhuzhou | 0.94043747 | 0.541669799 | 0.713727242 |
| 2019 | Ziyang | 0.918491109 | 0.439340094 | 0.635240088 |
| 2019 | Zigong | 0.934655092 | 0.48289958 | 0.671821816 |
| 2019 | Zunyi | 0.994237387 | 0.588887278 | 0.765175632 |
