# Supplementary material for: Research on coupling coordination and influencing factors between Urban low-carbon economy efficiency and digital finance—Evidence from 100 cities in China’s Yangtze River economic belt
Source: PLoS One. 2022 Jul 29;17(7):e0271455. doi: 10.1371/journal.pone.0271455 (PMC9337701; doi:10.1371/journal.pone.0271455)
Supplement: Attachment — Submitted filename: Response to Reviewers.docx [file pone.0271455.s004.docx]

Dear Editors and Reviewers,

Thank you very much for your kindly comments concerning our manuscript entitled “Research on Coupling Coordination and Influencing Factors between Urban Low-carbon Economy Efficiency and Digital Finance -- Evidence from 100 Cities in China's Yangtze River Economic Belt.” (PONE-D-22-00910R1). Your comments and those of the reviewers are all valuable and very helpful for revising and improving our paper, as well as the important guiding significance to our researches. We have studied comments carefully and have made correction which we hope meet with approval. Revisions in the text are shown using yellow highlight [example] for additions, and strikethrough font [~~example~~] for deletions. We hope that the revisions in the manuscript and our accompanying responses will be sufficient to make our manuscript suitable for publication in *PLOS ONE.* The main corrections in the paper and the responds to the reviewer’s comments are as flowing.

**Responds to the Journal’s comments:**

1. **Please review your reference list to ensure that it is complete and correct. If you have cited papers that have been retracted, please include the rationale for doing so in the manuscript text, or remove these references and replace them with relevant current references. Any changes to the reference list should be mentioned in the rebuttal letter that accompanies your revised manuscript. If you need to cite a retracted article, indicate the article’s retracted status in the References list and also include a citation and full reference for the retraction notice.**

**Response:** We fully respect the requirements of the journal. We have checked the reference list for the latest revised manuscript and confirmed that it is complete and correct. We have not cited a retracted article as a reference. We have made the following changes to the reference list, we have added a new reference (DOI: 10.3389/fenvs.2022.886886), added page numbers to some references (DOI: 10.1016/j.techfore.2020.120485; DOI: 10.3390/ buildings12010083), and the DOI of one reference was corrected (DOI: 10.15666/aeer/1705_1224512258).

**Responds to the additional editor’s comments:**

1. **The reviewers have provided comments for further revision of this manuscript, please revise in accordance with these comments.**

**Reponse:** We humbly accepted the additional editor’s comments, fully considered the comments of reviewer #2. In accordance with the reviewers’ suggestion, we have made the following main changes to the article:

1. We have improved the clarity of the images. We increased the resolution of the images and checked them using the PACE tool recommended by the journal to ensure they meet the journal's requirements.
2. We have revised the discussion section of the article into a separate section as suggested by the reviewer.

**Responds to the reviewer’s comments:**

**Responses to the comments of reviewer #1**

1. **Accept as it is
   Accept as it is
   Accept as it is
   Accept as it is
   Accept as it is
   Accept as it is
   Accept as it is**

**Response:** We are very grateful to reviewer #1 for his review suggestions, which have been very helpful in improving the article.

**Responses to the comments of reviewer #2**

We highly appreciate reviewer #2 for his insightful comments and criticism, which have helped us improve both the content and the presentation of our work.We have revised our manuscript, according to the reviewers’ comments and suggestions.

1. **Figures need to improve clarity.**

**Response:**We appreciate the reviewers' comments, and we have improved the clarity of the images. We upgraded the resolution of each image and checked the images using the journal's recommended PACE tool to ensure they met the journal's requirements. We have found that viewing the images directly through the PDF file generated by the submission system may not allow you to see the images clearly because they are compressed. We recommend that reviewer #2 download the images by clicking on the link at the top right of the images in the PDF and then view them, as this will ensure that the images are clear. Adjusted image, as follows：

Figure 1. Trend of ULEE

Figure 2. Spatial distribution of ULEE

Figure 3. Trend of the CCD

Figure 4. Evolution of the CCD

Figure 5. Spatial distribution of the CCD

Figure 6. The changes of relevant parameters of SDE

Figure 7. Forecast of spatial pattern

2. **It is recommended that the Discussion section be a separate chapter.**

**Response:** We are very grateful for the comments made by reviewer #2 and we have revised the Discussion section of the paper into a separate section. In the Disscusion section, we explain the findings of the article and answer the research questions posed in the Introduction section. We also further elaborate on the significance of this study in relation to previous research and explain the limitations of this study in Disscusion section. In addition, we have added a reference in Disscusion section (DOI: 10.3389/fenvs.2022.886886).

The Discussion section of the revised manuscript reads as follows:

This paper measured the ULEE of 100 cities in the YEB region of China and then analyzed the spatial and temporal evolutionary trends of the coupled coordination of ULEE and digital finance and the changes in the main influencing factors in conjunction with the Peking University Digital Inclusive Finance Index. These findings can help policy research departments to understand the coordinated development relationship between ULEE and digital finance in the YEB region of China in recent years, to better promote the two systems to achieve quality and harmonious development.

First, from a time evolution perspective, ULEE showed an overall fluctuating growth trend from 2012-2019, with a total increase of 7.41%. On the one hand, PEC and SEC grown by 13.16% and 4.8% respectively, indicating that the growth of ULEE is mainly driven by PEC and SEC. This is because the Chinese government has formulated a set of policies to boost the growth of the low-carbon economy since 2012 to encourage enterprises to use clean energy and improve energy use efficiency, which has led to the widespread and in-depth use of carbon reduction technologies in cities in the YEB region (Lin et al., 2021). On the other hand, the TC declined, which also indicates that the lack of science and technology innovation in the YEB region has limited the further progress of the efficiency of low-carbon economic development in the region. However, the TC has shown a significant increase in 2015, which may be attributed to the Chinese government's enhanced financial support and further implementation of tax breaks for low-carbon industries in 2015, which has boosted low-carbon technological innovation in enterprises (Hadfield et al., 2019). Therefore, promoting low-carbon production technology innovation in the R&D sector and developing new clean energy sources will be an important direction for the growth of the low-carbon economy in cities in the YEB region in the future. In addition, ULEE started to show continuous and stable growth after 2017, which may be related to the official launch of the carbon emission trading market at the end of 2017. Under the market mechanism, market players with excess or insufficient allowances can meet their emission reduction targets more effectively through trading. In other words, the carbon trading mechanism reduces the cost of carbon reduction for enterprises and accelerates the innovation and diffusion of low-carbon technologies (Lyu et al., 2020), thus promoting the optimization of industrial structure and realizing the efficiency of the low-carbon economy. From the perspective of spatial distribution, the upstream region had the highest ULEE, which mainly benefited from the efficient application of low-carbon technologies and the spillover of scale dividends, but there is still room for improvement in technological innovation in this region. The midstream region had the lowest PEC among the three regions, and its low-carbon economy was at the scale development stage. Therefore, the midstream region should accelerate low-carbon technology innovation and strengthen the application and diffusion of low-carbon technologies. The downstream region outperformed the other regions in terms of low carbon technology innovation, but had the lowest level of SEC, indicating that the downstream region had the relatively least scale dividend. From the perspective of city characteristics, Yuxi, Jingzhou, Zunyi, Lijiang, Qujing, Guangyuan, Changzhou, and Shanghai had high levels of ULEE among the YEB regions in China. Among them, Yuxi, Jingzhou, Zunyi, Lijiang, Qujing, and Guangyuan, inspired by China's high-quality development goals, have rapidly developed low-carbon tourism and significantly increased their fiscal revenues, thus implementing a set of policies to boost energy restructuring, including rational use of natural superiorities to exploit low-carbon energy, improve energy use efficiency and vigorously promote low-carbon lifestyles to the public, which have greatly enhanced the cities' ULEE. As for Changzhou and Shanghai, both cities have relatively sound infrastructure, reasonable industrial structures, convenient transportation, a concentration of talents, and a strong capacity for low-carbon technological innovation. Shanghai, in particular, as the world's financial center, has a high level of digital financial development, which offers efficient financial support for the development of green technologies and is contributive to the study, development, and promotion of new clean energy sources, which can significantly improve the city's ability to reduce carbon emissions. The ULEE of Zhaotong, Taizhou, Ziyang, Huzhou, Jiaxing, Dazhou, Changsha, Fuzhou, Nanchang, Yichang, Wenzhou, Liu'an, Loudi, and Jingmen was at a relatively low level in the YEB zone, primarily due to the low level of TC in these cities, and therefore the innovation and application of low-carbon technologies should be strengthened. The low PEC in Xianning and Yichun restricted the improvement of ULEE, so optimizing the application of technology and improving the management of technology should be the focus of future development in these cities. The mean values of SEC in Ji'an and Jingmen were less than 1. They need to expand their production scale and increase factor inputs for low-carbon economic development, to improve ULEE. In summary, this paper here discussed the spatio-temporal evolution characteristics of ULEE in China's YEB region from 2012-2019, answering the first question raised in Section 1.

Second, from the perspective of time evolution, CCD showed an overall trend of stable growth from 2012-2019. Specifically, the CCD grew slowly from 2012-2016. This is mainly because digital finance was still in its infancy and the intensity of support for the growth of the low-carbon economy was insufficient, resulting in slow growth of ULEE and thus slowing down the coordinated development of ULEE and digital finance (Zhao et al., 2021). CCD has continued to grow after 2017, probably due to the rapid growth of China's digital finance sector and the launch of China's carbon emission rights market, which have stimulated the advance of low-carbon industries and the restructuring of energy consumption, promoting urban carbon reduction (Tan et al., 2022), thus allowing for a higher level of coordination between the two systems. From the perspective of spatial evolution, most of the cities in the YEB experienced a shift from approaching imbalance and slight imbalance to primary coordination and intermediate coordination during the study period, which indicates that the coordinated development relationship between ULEE and digital finance in the YEB region has been widely improved. Among them, the lower Yangtze River region had the highest degree of digital finance development relative to other regions, allowing cities to receive more abundant and efficient financial support in the process of low-carbon economic development, thus promoting clean technology research and development and improving the energy consumption structure to achieve a high level of CCD development. The cities in the upstream region are mostly inland cities, where access to resources such as labor, technology, and capital is difficult, and production and operation are mostly based on crude industrial production, making it difficult for the urban economy and urban environment to develop in a coordinated manner (Fang et al., 2021). Meanwhile, the growth of digital finance in the region is comparatively lagging, with insufficient financing support, making it hard for the two to accomplish coordinated evolution, and therefore the CCD level in the region was the lowest. The value of the CCD in the middle reaches of the Yangtze River lies between the CCD in the upper reaches and those in the lower reaches. In addition, the cities in the YEB region with a relatively high level of coordinated development of ULEE and digital finance are mainly located in the northeast-southwest direction. The spatial center of gravity shifted first to the northeast, then to the northwest, and finally to the southwest during the study period. This is mainly because the CCDs of cities such as Liu'an, Zhenjiang, and Yancheng, which are located in the northeast direction, showed a significant increase from 2012-2014. CCDs in cities such as Mianyang and Chengdu, located in the northwest direction, increased significantly from 2014-2016. CCDs in cities in the southwest of the YEB increased at a faster rate after 2016, while CCDs in cities in the northeast maintained a relatively low rate of growth. Thus, a trajectory of the center of gravity moving first to the northeast, then to the northwest, and finally to the southwest was formed. In terms of the future direction of the spatial pattern, the spatial center of gravity of CCD will move southwestward in the middle region of the YEB in 2019-2040, indicating that the western cities in the middle Yangtze River region are expected to boost the coordinated growth of ULEE and digital finance in the future. From 2019 to 2040, the discrete level of CCD in the northeast-southwest direction will show a downward trend, while the discrete level in the northwest-southeast direction will increase, indicating that cities in the northwest-southeast direction have the possibility of rapidly increasing CCD, and the exchange of low-carbon technologies, financial resources, digital technologies and technical talents between regions will be further strengthened, thus benefiting cities with relatively low CCD levels. In addition, the spatial pattern of CCD in 2019-2040 is dominated by the northeast-southwest direction and will turn in a counterclockwise direction, again indicating that CCD in cities with a northwest-southeast direction has a strong potential to improve in the future. In summary, this paper here discusses the spatio-temporal evolutionary characteristics of the CCD of ULEE and digital finance from 2012-2019 and the future spatial pattern changes, answering the second question posed in Section 1.

Third, in terms of influencing factors, the level of informatization and industrial structure tended to gradually increase their influence on CCD, while the level of population development, greening, transportation, and scientific research gradually weaken their influence on CCD. In terms of the influence of the level of population development, the influence of population development showed a slow downward trend overall, with an average annual decrease of 0.35%. However, the influence of this factor was still at a relatively high level compared to other factors. This is mainly because as the number of people increases, there is a corresponding increase in social labor resources and consumption behavior of the population, which helps to boost the efficiency of economic development while promoting the development of the digital financial sector. Changes in low-carbon consumption behavior would also have a vital influence on carbon emissions. As a result, the influence of the level of population development on CCD was slowly diminishing but still at a high level. In terms of the impact of the level of informatization, the impact of the level of informatization showed an overall trend of growth over time, with an average annual growth of 1.17%. This is because the popularity of the Internet is the basis for the creation and advance of digital finance, and digital financial services need to be realized through the Internet and related information technology. And the construction of information technology has given impetus to the birth of more new green industries, providing new impetus to the growth of the low-carbon economy. In terms of the impact of greening levels, the overall impact of greening levels showed a slow downward trend, with an average annual decrease of 0.33%. This may be because with the deepening of greening construction, the space available for greening construction in built-up areas gradually becomes less and the marginal contribution of greening level to the low-carbon economy gradually decreases, and the driving effect of greening construction on the low-carbon economy diminishes, thus obstructing the coordinated advance of ULEE and digital finance. In terms of the influence of transport levels, the influence of this influence showed an overall decreasing trend, with the influence increasing year on year from 2012-2017, but decreasing sharply after 2017, which should be attributed to the increase in the number of public-operated trams from 2012-2017, the increase in the number of low-carbon trips made by urban residents and the corresponding increase in urban transport capacity, which led to a good development of urban low-carbon economic efficiency, but in recent years the number of public-operated trams in the city gradually saturated and the number of private cars in the city increased sharply, boosting a raise in CO2 emissions. In terms of the influence of industrial structure, the influence of this factor on CCD increased gradually, indicating that the Chinese government issued relevant policies to promote the proportion of tertiary industries in GDP, eliminating industries related to high pollution, high energy consumption, and high carbon emissions, and services such as digital finance have been better developed, and further improvement of industry construction is an important direction to coordinate the development of ULEE and digital finance. In terms of the influence of the level of scientific research, the influence of the level of scientific research on CCD decreased overall, but its influence was still at a high level compared to other factors, which indicates that improving the level of scientific research and innovation is still a priority for the development of cities in the YEB region of China and that cities should actively accelerate the upgrading of energy conservation and digital information technology, while ensuring that cities make effective use and promotion of existing technology, fully applying advanced technology to the growth of low carbon economies in cities, and accelerating the construction of digital financial services so that digital finance can provide strong financial support for the growth of the low carbon economy in the future. In summary, this paper discussed here the impact of factors such as level of population development, level of informatization, level of greening, level of transportation, industrial structure, and level of scientific research on the CCD of ULEE and digital finance in the YEB region of China from 2012-2019, answering the third question posed in Section 1.

Besides the above answers to the research questions, this paper provided several novel contributions to related studies. Firstly, in terms of ULEE evaluation, this paper complemented the framework for evaluating low-carbon economic efficiency in previous studies (Meng et al., 2018; Zhang et al., 2017). Compared with existing researches, this paper improved the evaluation framework of low carbon economy efficiency in terms of land use and financial support. Other studies on low-carbon economy efficiency can be further expanded based on this, such as the interaction between low-carbon economy efficiency and the level of technological development (Yang et al., 2022). Secondly, this study provided a new quantitative analysis perspective and is the first study to analyze the changes in the level of coordinated development of ULEE and digital finance through a coupled coordination degree model, which helps to help relevant sectors understand the coordinated development relationship between ULEE and digital finance. Thirdly, this paper further analyzed the expected changes in the spatial pattern of CCD between ULEE and digital finance and the influencing factors, which will provide a basis for local governments in China to formulate policies to enhance the level of coordinated development of ULEE and digital finance.

In addition, there are still some limitations to this paper. Firstly, the paper uses the latest data that can be collected at present, but there is still a certain lag, for further research can be done after the data is updated. Secondly, this paper does not take into account the climate change factor in the ULEE evaluation framework, and the model can be expanded in future research to improve the ULEE evaluation framework. Thirdly, this paper only considers the effect of individual factors on the level of coordinated growth of ULEE and digital finance but does not analyze the interaction of multiple influencing factors, which can be discussed in more depth in future research.

Thank you again for your positive and constructive comments and suggestions on our manuscript. We have tried our best to improve the manuscript and made some changes in the manuscript. These changes will not influence the framework of paper.

We appreciate for Editors and Reviewers’ warm work earnestly, and hope the correction will meet with approval.

yours sincerely,

Fengge Yao, Liqing Xue, Jiayuan Liang

Corresponding author:

Name: Jiayuan Liang

E-mail:timljy1994@gmail.com
